# JP1 suppresses proliferation and metastasis of melanoma through MEK1/2 mediated NEDD4L-SP1-Integrin αvβ3 signaling

**DOI:** 10.7150/thno.45843

**Published:** 2020-07-01

**Authors:** Jiahua Cui, Chuanjun Shu, Jin Xu, Dongyin Chen, Jin Li, Kun Ding, Minjuan Chen, Aiping Li, Jingdong He, Yongqian Shu, Liuqing Yang, Ruiwen Zhang, Jianwei Zhou

**Affiliations:** 1 Department of Molecular Cell Biology & Toxicology, Center for Global Health, School of Public Health, Nanjing Medical University, Nanjing 211166, China; 2 Jiangsu Key Lab of Cancer Biomarkers, Prevention and Treatment, Collaborative Innovation Center for Cancer Medicine, Nanjing Medical University, Nanjing 211166, China.; 3 Department of Bioinformatics, School of Biomedical Engineering and Informatics, Nanjing Medical University, Nanjing 211166, China; 4 Department of Medicinal Chemistry, School of Pharmacy, Nanjing Medical University, Nanjing 211166, China; 5 Department of Oncology, the Affiliated No. 1 Hospital of Nanjing Medical University, Huaian, Jiangsu Province, China.; 6 Department of Oncology, the First Affiliated Hospital of Nanjing Medical University, Nanjing 210029, China.; 7 Department of Molecular and Cellular Oncology, the University of Texas MD Anderson Cancer Center, Houston, TX, USA.; 8 Department of Pharmacological and Pharmaceutical Sciences, College of Pharmacy, University of Houston, Houston, TX, 77204, USA.

**Keywords:** melanoma, therapeutic peptide, NEDD4L, SP1, integrin αvβ3.

## Abstract

**Background:** JWA gene is known to down-regulate SP1 and reduces the expression level of Integrin αvβ3. Here, we identified a functional polypeptide (JP1) based on the active fragment of the JWA protein to suppress melanoma growth and metastasis by inhibiting the Integrin αvβ3.

**Methods:** We conducted a series of melanoma growth and metastasis mouse models to evaluate anti-melanoma effect of JP1 peptide. ^18^F-labeled JP1 (^18^F-NFP-JP1) was detected by Micro-PET assay to demonstrate drug biodistribution. Toxicity test in cynomolgus monkeys and pharmacokinetic studies in rats were done to assess the druggability. The expression of MEK1/2, NEDD4L, SP1 and Integrin αvβ3 were detected *in vitro* and *vivo* models.

**Results:** The peptide JP1 with the best anticancer effect was obtained. Micro-PET assay showed that JP1 specifically targeting to melanoma cells *in vivo*. JP1 inhibited melanoma growth, metastasis, and prolonged the survival of mouse. JP1 reduced the dosage and toxicity in combination with DTIC in melanoma xenograft and allograft mouse models. Cynomolgus monkey toxicity test showed no observed adverse effect level (NOAEL) of JP1 was 150 mg/kg. Mechanistically, JP1 was shown to activate p-MEK1/2 and triggered SP1 ubiquitination in melanoma cells. NEDD4L, an E3 ubiquitin ligase, was activated by p-MEK1/2 and to ubiquitinate SP1 at K685 site, resulting in subsequent degradation.

**Conclusions:** JP1 was developed as a novel peptide that indicated therapeutic roles on proliferation and metastasis of melanoma through the NEDD4L-SP1-Integrin αvβ3 signaling.

## Introduction

Melanoma is a deadly form of malignancy 1. In the United States, although melanoma accounts for only 2% of all skin-related cancers, it causes 80% of deaths from dermatological cancer 2. Immunotherapy has made a breakthrough in the early treatment of melanoma, significantly improved the progression-free survival of patients 3. However, immunological checkpoint inhibitors relieve the immune system's inhibitory state, and related adverse events involved several organs and systems 4. The drugs targeting melanoma BRAF mutations are also posing a major challenge to effective long-term cancer clinical treatment 5. Even when inhibitors produce significant effects at an early stage, acquired resistance subsequently comes 6, 7. The increase in drug-resistant cancer cells limits the available treatment options. Therefore, the development of novel drugs to effectively resist melanoma and prolong the progression-free survival of patients is urgent needs.

Integrin αvβ3 is critical for the transformation of extracellular signals into intracellular responses, particularly in acting as transmembrane link between the extracellular matrix and the intracellular actin backbone 8. However, it is widely expressed on the membrane surface of melanoma and associated with tumor angiogenesis, cell migration and proliferation, and metastasis 9. Integrin αvβ3 targeted therapeutic drugs including therapeutic antibody-based drugs, peptide-based drugs and small molecule-based drugs has conducted clinical trials, however none of them available used in clinical therapy 10. Integrin αvβ3 as an anticancer target has broad application prospects.

Peptide drugs are widely used due to relative safe and remarkable curative action and have gained much interest as anticancer agents 11, 12. Peptide therapeutics are typically associated with lower production complexity compared with protein-based biopharmaceuticals 13. Rational design of peptide is usually started with functional fragments of a protein and may be optimized by additional necessary modifications in its structure 14.

JWA gene, also known as ADP ribosylation factor like GTPase 6 interacting protein 5 (ARL6IP5), is initially cloned by Zhou et al from retinoic acid induced HBE cell differentiation models 15. Studies have shown that JWA is a cytoskeleton binding protein and functionally involved in oxidative stress responsiveness 16, DNA repair 17, cell migration 18 and angiogenesis 19, 20. Further evidences indicate that JWA exerts tumor suppressor function in several cancers including melanoma 20, 21. The mechanistic studies show that JWA conducts its anticancer functions partly via MAPK dependent pathway 18, 22. Interestingly, unlike other MAPK signaling inhibitors 23, JWA degrades overexpressed oncoproteins or its transcription factors by MAPK pathway mediated activations of E3 ubiquitin ligases. Therefore, JWA works as an agonist to MAPK signal pathway, and then as an inhibitor of oncoproteins through E3 ubiquitin ligase linked action mode.

In this study, we obtained an anticancer peptide JP1, which was designed and optimized a seven amino acids fragment of JWA protein, with pre-phosphorylation and target integrin αvβ3. Using cell and animal models, we not only determined the anti-proliferation and anti-metastasis effects of JP1 on melanoma, but also elucidated its molecular mechanisms of action. In particular, we have completed a series of studies on the druggability of JP1, demonstrating its value as a novel potential therapeutic anticancer agent.

## Results

### Designing and screening of JP1 anti-melanoma peptide in mouse models

**Figure 1:**
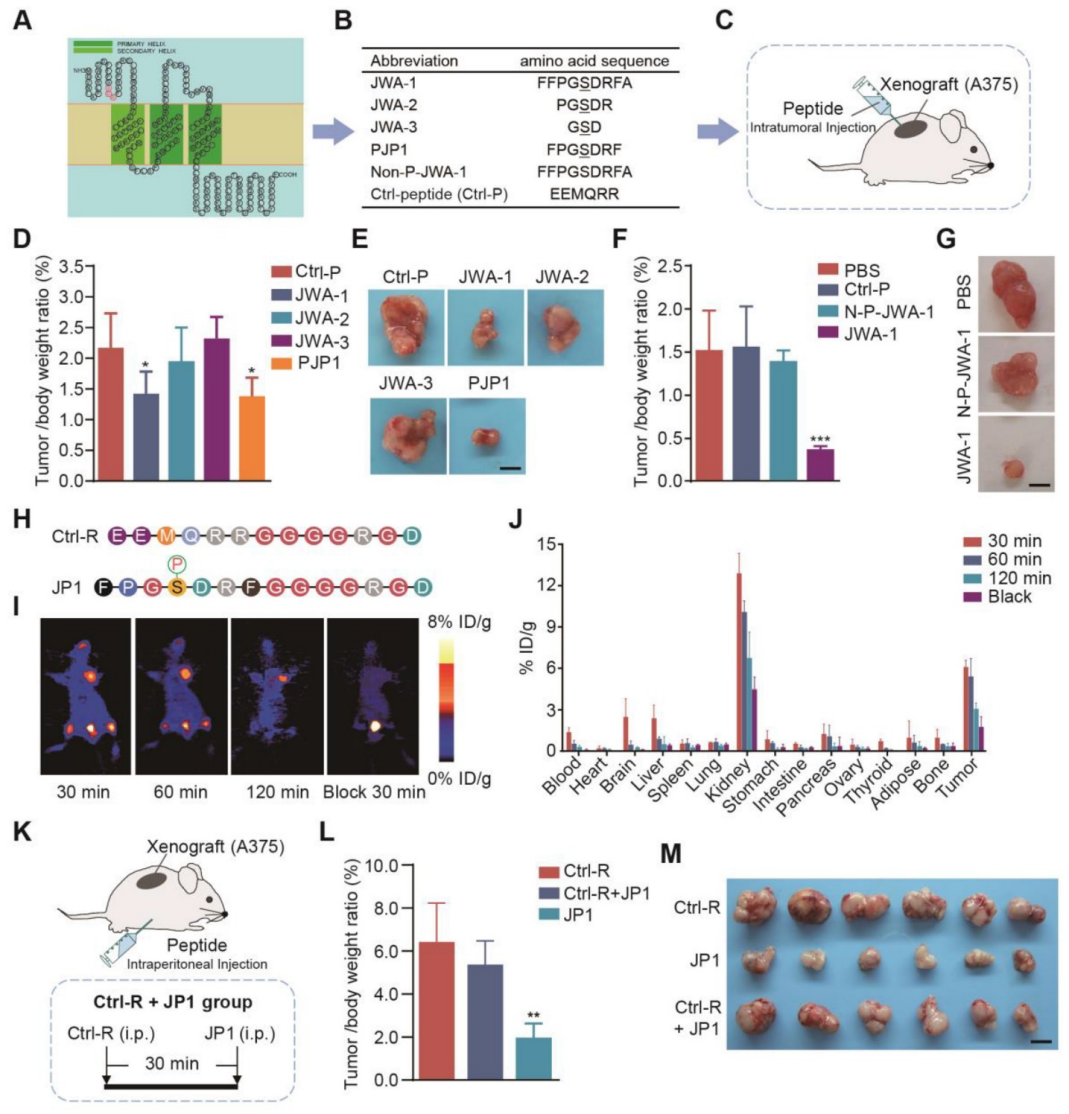
** Screening of a highly selective anticancer JWA peptide. (A)** The predicted JWA protein structure; the SDR motif was labeled as red; three transmembrane domains were shown in green. **(B)** The sequence of JP1 candidate fragments; the serine-phosphorylated peptides with different lengths and non-phosphorylated JWA-1 peptide (Non-P-JWA-1); and non-sense control (Ctrl-P). **(C)** The diagram of A375 melanoma tumor-bearing model for peptide screening. **(D-E)** The tumor inhibitory effect of peptides (n = 6 per group): **(D)** The tumor/body weight of peptides were shown. **(E)** Representative images of subcutaneous tumors for the indicated treatments. Scale bar, 1 cm. *P < 0.05.** (F-G)** The tumor inhibitory effect of peptides with phosphorylated or non-phosphorylated modifications (n = 6 per group): **(F)** The tumor/body weight of peptides with phosphorylated or non-phosphorylated modifications.** (G)** Representative images of subcutaneous tumors for indicated treatments. Scale bar, 1 cm. ***P < 0.001.** (H)** The highlighted Ctrl-R sequence and JP1 sequence with modifications. **(I-J)** The representative PET images and biodistribution of ^18^F-NFP-JP1 in A375 xenografted tumor-bearing mouse:** (I)** Representative PET images of ^18^F-NFP-JP1 at 30, 60, and 120 min after injection. **(J)** The biodistribution of ^18^F-NFP-JP1 in A375 xenografted tumor-bearing mouse.** (K-M) (K)** The diagram of A375 melanoma tumor-bearing nude mouse model for targeting peptide screening (n = 6 per group). **(L)** The tumor/body weight of the indicated treatments. **(M)** The representative images of subcutaneous tumors for indicated treatments. Scale bar, 1 cm. **P < 0.01.

Previous studies have shown that phosphorylation of serine site in SDR motif of JWA protein sequences is necessary for JWA mediated activation of MAPK signaling and inhibition of tumor cell migration 18. To identify the potential functional fragments of JWA protein, we first predicted the structure of JWA protein and designed the fragments containing SDR sequences for functional screening **(Figure 1A)**. We next designed a series of serine-phosphorylated peptide fragments with different lengths (3-, 5-, 7- and 9-amino acid fragments with a core amino acid serine) and observed the anti-proliferation effect of the peptides in human melanoma cell (A375) xenograft mouse model **(Figure 1B-1C)**. As a result, PJP1, which was contained seven amino acids, showed the best anti-proliferation activity compared with the fragments of other lengths **(Figure 1D-1E, Figure S1A-S1B)**. Further data showed the phosphorylation of the serine was necessary for the effects of PJP1 compared to the non-phosphorylated peptide (N-P-JWA-1) **(Figure 1F-1G, Figure S1C-S1D)**.

Next, we used a targeting strategy to overcome intrinsic problem of peptide targeting melanoma. It is well known that integrin αvβ3 is overexpressed in the membrane of melanoma cells and recognized by the amino acid triplet Arg-Gly-Asp (RGD) 24. The PJP1 peptide-linked RGD motif (named JP1) and the Ctrl peptide-linked RGD motif (named Ctrl-R) were then designed and used for experimental therapy in melanoma xenograft and metastatic mouse models **(Figure 1H)**. To determine tumor targeting of JP1, micro-PET imaging bind biodistribution studies were conducted after injecting (by tail vein) ^18^F-labeled JP1 (^18^F-NFP-JP1), and the results showed that ^18^F-NFP-JP1 specifically accumulated in the melanoma tumor mass, bladder, and femoral arteries on both sides of the thigh **(Figure 1I)**. Furthermore, treatment of xenograft mouse with a triple dose of Ctrl-R (non-sense peptide-linked RGD motif) at 30 min before ^18^F-NFP-JP1 injection completely blocked JP1 binding to melanoma tumor cells **(Figure 1I)**. As shown in **Figure 1J**, absorbed JP1 peptide was mostly distributed in both kidney and tumor mass. The tumor inhibitory effect of JP1 was confirmed in A375 cell xenograft mouse model **(Figure 1K)**. As shown in **Figure 1L**, JP1 indicated a significant anti-proliferation effect on A375 xenograft tumor compared to the Ctrl-R and the pre-blocked JP1 groups (*P* < 0.01). The isolated tumor mass and tumor growth curves were shown in **Figure 1M, Figure S1E-S1F**. Collectively, JP1 targeted and inhibited melanoma proliferation in xenograft mouse model.

### JP1 inhibits proliferation and metastasis of melanoma *in vivo*

**Figure 2:**
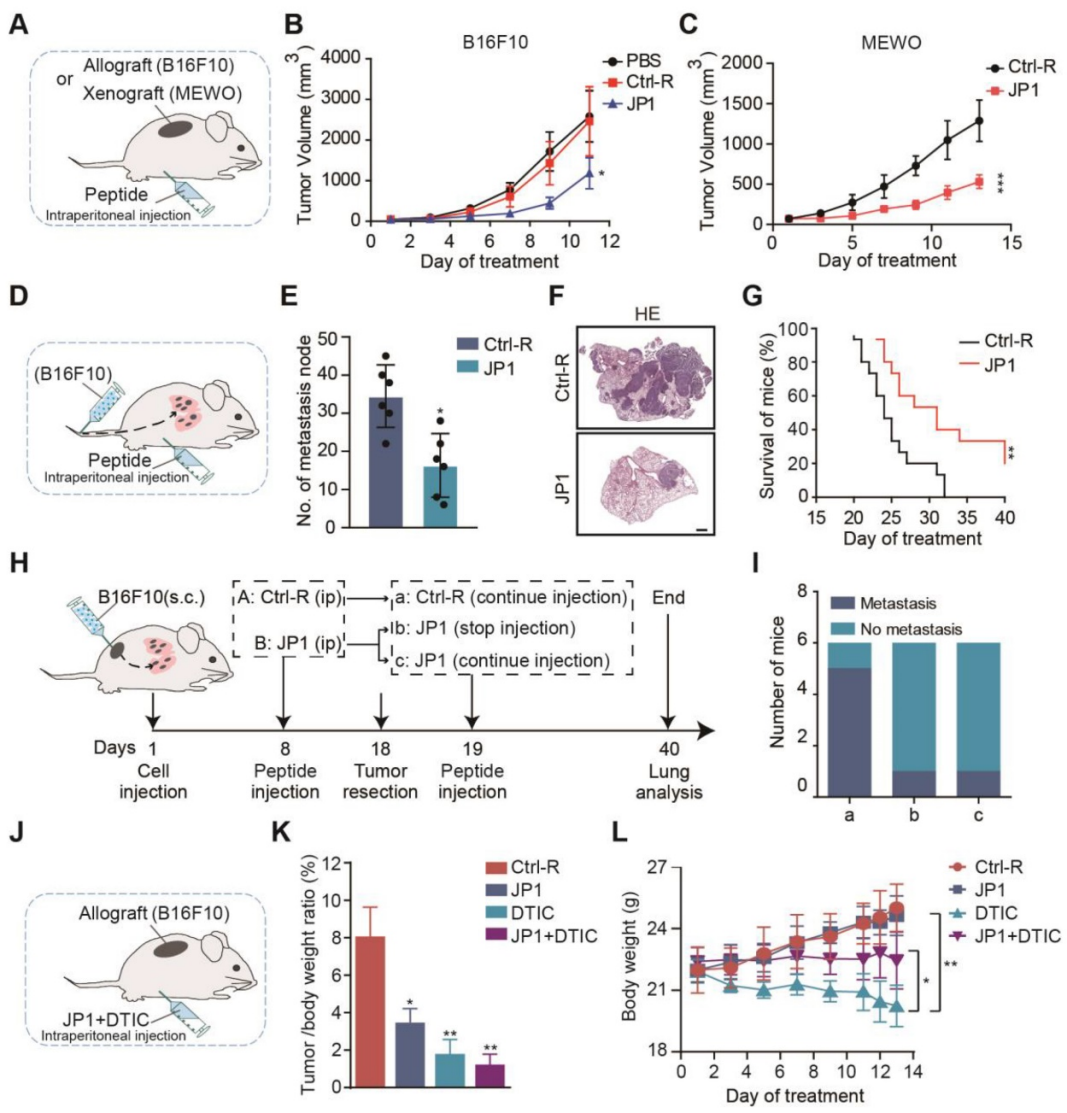
** JP1 inhibits growth and metastasis of melanoma *in vivo.* (A-C) (A)** Schematic representation of the B16F10 and MEWO cells melanoma-bearing model for JP1 treatment (n = 6 per group). JP1 and its control agents were administered by intraperitoneal injection.** (B)** The tumor growth curves of B16F10 cells injection in PBS, Ctrl-R or JP1 treated mouse. *P < 0.05.** (C)** The tumor growth curves of MEWO cells injection in Ctrl-R or JP1 treated mouse. ***P < 0.001.** (D-G) (D)** Schematic representation of the B16F10 cell melanoma passive metastasis model for JP1 treatment. JP1 and its control agents were administered by intraperitoneal injection. **(E)** Number of metastasis node per mouse were counted after Ctrl-R or JP1 treatments (n=6 per group). *P < 0.05. **(F)** The representative melanoma lung metastatic images by H&E-staining (scale bars, 1000 μm). **(G)** Kaplan-Meier survival curve after Ctrl-R or JP1 treatments (n = 15 per group). **P < 0.01.** (H-I) (H)** Schematic representation of the B16F10 cell melanoma allografts and followed active metastasis two-stage model for JP1 treatment. **(I)** Graph showed the number of mouse that developed lung metastases 3 weeks after surgical removal of the primary tumor with indicated treatments (n = 6 per group). **(J-L) (J)** Schematic representation of the B16F10 cell melanoma-bearing model (n= 6 per group). Both JP1 and DTIC alone or in combination treatments and relative control was administered by intraperitoneal injection in mice. **(K)** The tumor/body weight ratio of after indicated treatments. *P < 0.05, **P < 0.01. **(L)** The body weights at the indicated time points after indicated treatments. *P < 0.05, ** P < 0.01.

To determine the therapeutic values of JP1 on melanoma, we completed a series of mouse models. We first constructed B16F10 and MEWO cells melanoma-bearing mouse model **(Figure 2A)**. In B16F10 cells melanoma-bearing mouse model, compared with Ctrl-R, JP1 inhibited the growth of melanoma and with a tumor inhibition rate of 55% **(Figure 2B, Figure S2A-S2C)**. In MEWO cells melanoma-bearing mouse model, JP1 inhibited the growth of melanoma and with a tumor inhibition rate of 51% compared with Ctrl-R **(Figure 2C, Figure S2D-S2F)**.

We next evaluated the effect of JP1 by B16F10 cells melanoma metastasis mouse model. Melanoma passive metastasis model was conducted and JP1 was treated by intra-peritoneal injection **(Figure 2D)**. On the 21^st^ day of JP1 treatment, the model was ended and pulmonary metastasis in mouse was evaluated. As shown, the number of lung metastasis nodules were decreased by 53% in JP1 group compared with Ctrl-R group **(Figure 2E-2F, Figure S2G)**. Moreover, a parallel survival mouse model was conducted by B16F10 cells passive metastasis. As shown in **Figure 2G, Figure S2H-S2I**, compared to the Ctrl-R treated mouse, JP1 treatment effectively prolonged mouse survival (*P* < 0.01). To mimic the effects of JP1 anticancer in animal models close to clinical practice, we performed a two-phase melanoma cell allograft tumor resection-metastasis mouse model. In the first phase, mouse were treated with JP1 for 8 days after being inoculated with melanoma cells (B16F10), and the tumor was removed surgically on day 18. In the second phase, mouse were treated with or without JP1. At the end of the model on day 40, lung tumor metastasis was observed **(Figure 2H)**. As the end point of the model, 5 out of 6 mice in Ctrl-R group showed lung metastasis **(Figure 2Ia)**, whereas, lung metastasis was observed in only 1 out of 6 mice in JP1 group **(Figure 2Ic)**; importantly, lung metastasis was only occurred in 1 out of 6 mice even if stopped JP1 treatment after tumor removal **(Figure 2Ib)**. The body weight in the first phase and lung wet weight of mouse at the end point was higher in Ctrl-R group than both JP1 treated mouse (*P* < 0.05, **Figure S2J-S2L**). These results indicate that JP1 is effective in inhibiting melanoma growth and metastasis.

### JP1 reduces the dosage and toxicity in combination with DTIC

The chemotherapeutic agent dacarbazine (DTIC) is currently as the first line drug used to treat metastatic melanoma in clinic, and was reported to cause painful symptoms and acquired resistance, compromising patient quality of life 25. To determine the possibility as a combination therapy between DTIC and JP1, we completed a joint treatment of DTIC and JP1 for melanoma tumor-bearing model **(Figure 2J)**. As shown, 80 mg/kg DTIC treatment indicated more significant inhibitory effect on melanoma proliferation than that by 50 mg/kg JP1 **(Figure 2K, Figure S3A-S3C)**; however, the mouse in DTIC group lost body weight continuously during treatment **(Figure 2L)**. HE staining data showed significant degenerations in liver cells after DTIC treatment **(Figure S3D)** although the other organs did not show obvious injuries after treatments **(Figure S3E)**. Of concern, treatment mouse with 50 mg/kg JP1 combined with 40 mg/kg DTIC showed similar anticancer effects with 80 mg/kg DTIC alone** (Figure 2K, Figure S3A-S3C)**; importantly, the body weight curve in mouse with the combined treatments was stable and without obvious liver injury compared to the DTIC alone** (Figure 2L, Figure S3E)**. These findings suggest that JP1 reduces the dosage and toxicity of DTIC when JP1 in combination with DTIC treatment for melanoma.

To further evaluate the safety of JP1 and accelerate the progress of preclinical trials, in addition to testing LD_50_ value of JP1 was greater than 5000 mg/kg in mouse, a 14-day cynomolgus monkey toxicity test was completed by a contract service in Suzhou Xishan Zhongke Drugs Research and Development Co., Ltd. Data showed that compared to the control group (by saline), the monkeys received JP1 (I.V.) either 45 or 150 mg/kg/day for 14 days had no significant changes in all detected parameters, including clinical symptoms, weight, food intake, body temperature, ophthalmology, electrocardiogram, blood routine examination, blood biochemistry, blood coagulation, urinalysis, immune-toxicity **(Figure S4, Supplementary database files: Cynomolgus monkey toxicity test)**. These results suggest no adverse effects were observed when JP1 was given in large doses for 14 days in healthy experimental monkeys.

### JP1 mediates degradation of SP1 by the ubiquitin-proteasome pathway

**Figure 3:**
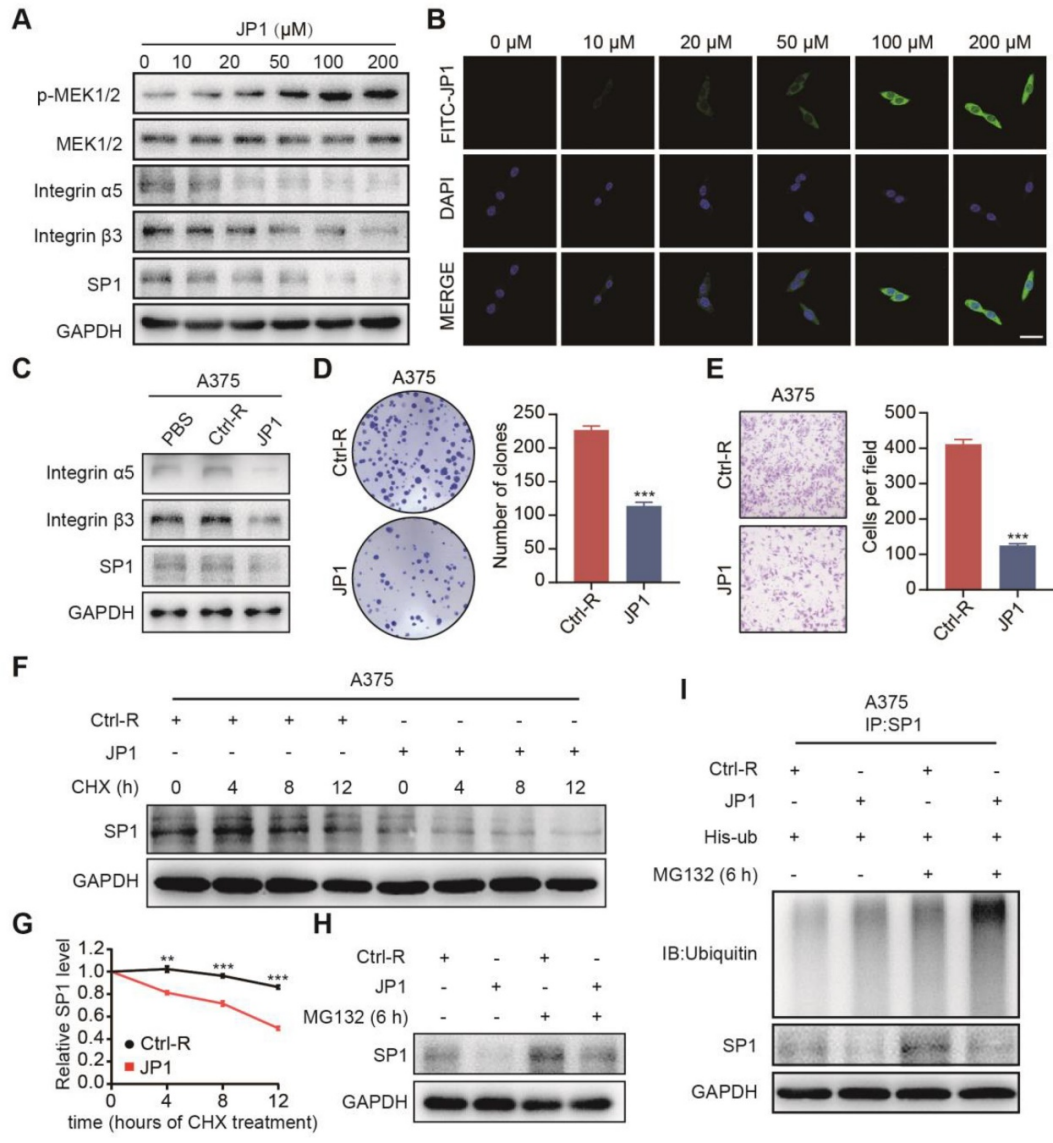
** JP1 mediates degradation of SP1 by the ubiquitin-proteasome pathway. (A)** A375 cells were treated with JP1 for indicated doses for 48 h. Immunoblotting showed the expression levels of indicated molecules. **(B)** B16F10 cells were treated with FITC-JP1 at indicated doses for 3 h. Immunofluorescence analyzed the intracellular distribution after treatment of FITC-JP1 (scale bars, 50 μm). **(C)** Immunoblotting analyzed the expression levels of integrin α5, integrin β3, SP1 after treatments of PBS, Ctrl-R, and JP1 in A375 cells. **(D-E)** The representative images and quantitative data of Colony formation **(D)** and transwell assays **(E)** of A375 cells, respectively. n = 3; ***P < 0.001. **(F-G) (F)** SP1 stability assay. The A375 cells were treated by JP1 for 48 h and followed by exposed to CHX for indicated time; expression of SP1 was determined by Immunoblotting. **(G)** The time-course intensities of the SP1 protein (n = 3). **P < 0.01, ***P < 0.001. **(H)** SP1 ubiquitination degradation assay. The A375 cells were treated JP1 for 48 h followed by MG132 for 6 h; SP1 was determined by Immunoblotting. **(I)** JP1 increases ubiquitination in SP1. The A375 cells were treated with JP1, followed by MG132; ubiquitinations and expressions of SP1 was determined by Co-IP and immunoblotting, respectively.

JWA gene has been demonstrated an up-stream molecule to activate MEK1/2, resulting in the subsequent reduction of melanoma proliferation and metastasis. Meanwhile, the role of JWA gene on melanoma is SP1 ubiquitination mediated down-regulation of integrin αvβ3. To confirm whether the roles of JP1 anti-melanoma was by a similar mechanism like JWA gene, we completed mechanistic investigations. As shown in **Figure 3A**, JP1 activated expressions of p-MEK1/2 while inhibited SP1 and integrin αvβ3 dose-dependently in human A375 melanoma cells. Similar results were obtained in JP1 treated melanoma tissues from the allograft and metastatic mouse models **(Figure S5A-S5B)**. The immunofluorescence results showed FITC-JP1 was distributed in cytoplasm and with a dose-dependent intensity in A375 cells in 3 h of exposure; and the intensity seemed to reach saturation at the 100 μM **(Figure 3B)**. JP1 inhibited expressions of integrin αvβ3 and SP1 in both A375 **(Figure 3C)** and MEWO melanoma cells **(Figure S5C)** compared to the cells treated by Ctrl-R or PBS. To determine the anticancer activity of JP1 *in vitro*, we performed colony formation and transwell assays in A375 and MEWO cells. The results showed that JP1 effectively inhibited cell proliferation and migration compared to the Ctrl-R treated cells** (Figure 3D-3E, Figure S5D-S5E)**.

To confirm the mechanism of how JP1 inhibits SP1, we determined the mRNA level of SP1 after JP1 treatment. The result showed that SP1 mRNA level was not affected by JP1 treatment **(Figure S5F)**. Next, we determined endogenous SP1 expression after exposure to JP1 and cycloheximide (CHX), an inhibitor of protein synthesis, in A375 and MEWO cells. As expected, the endogenous SP1 protein was decreased after exposure to CHX. Moreover, the degradation of SP1 was increased by JP1 treatment **(Figure 3F-3G, Figure S5G-S5H)**. When the cells were treated with MG132, a protease inhibitor, the degradation of SP1 was partly blocked **(Figure 3H, Figure S5I)**. These results were also confirmed in His-ub transfected A375 cells, the ubiquitination level of SP1 was increased after MG132 treatment and further strengthened by JP1 (**Figure 3I**). These data suggest that JP1 mediated degradation of SP1 by the ubiquitin-proteasome pathway.

### JP1 ubiquitinates SP1 by E3 ubiquitin ligase NEDD4L

**Figure 4:**
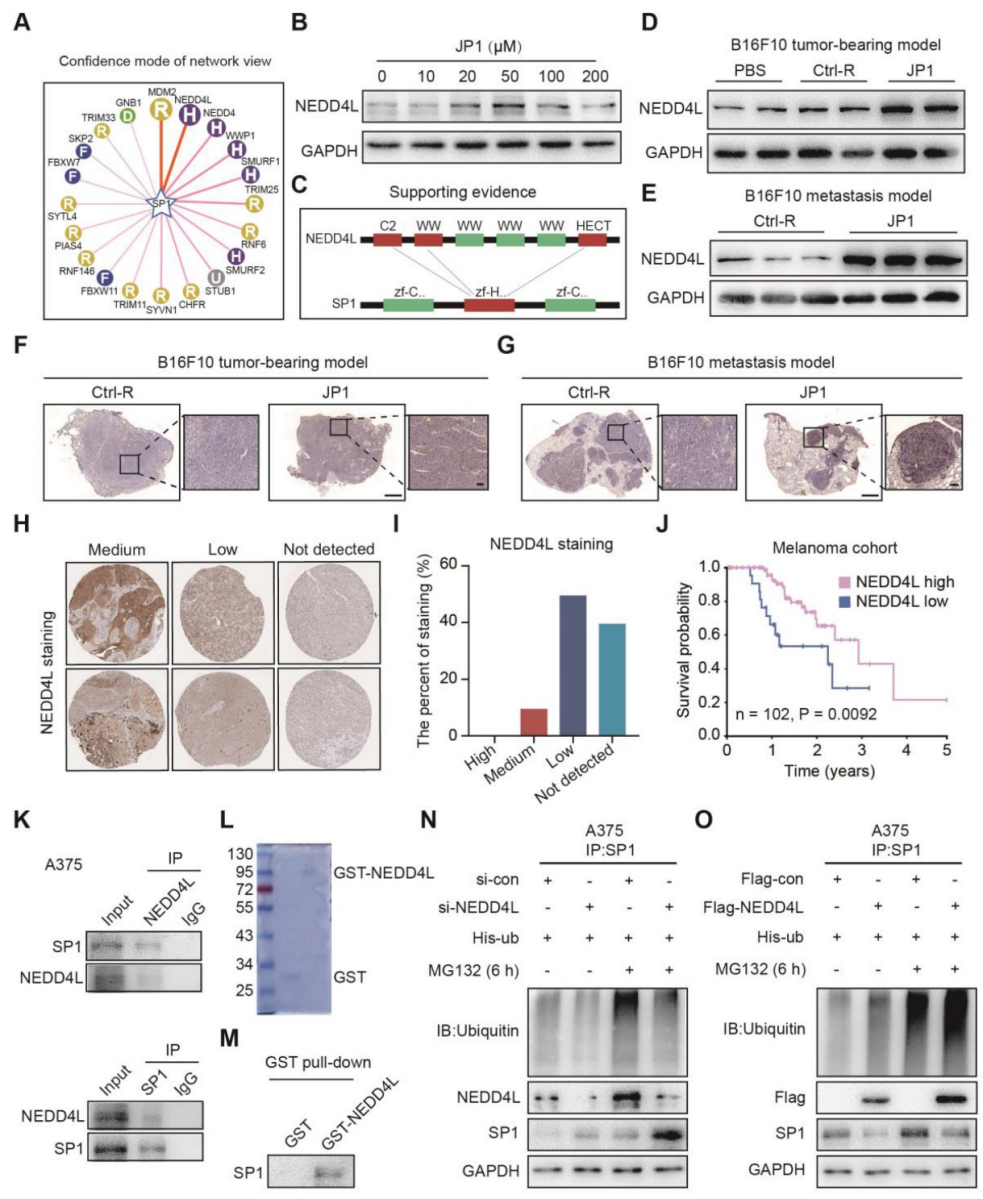
** JP1 ubiquitinates SP1 by E3 ubiquitin ligase NEDD4L. (A)** The predicted E3 ubiquitin ligases of SP1. **(B)** JP1 increases expression of NEDD4L in A375 cells, Immunoblotting data. **(C)** The schematic diagram of predicated interactions between NEDD4l and SP1. **(D-E)** Analysis of the expressions of NEDD4L in tumor tissues of melanoma tumor-bearing **(D)** and passive metastasis mouse models **(E)** by Immunoblotting. **(F-G)** Analysis of the expressions of NEDD4L in tumor tissues of melanoma tumor-bearing **(F)** and passive metastasis mouse models **(G)** by IHC assay (scale bars, 1000 μm and 100 μm, respectively). **(H-I)** Analysis based on the database of The Human Protein Atlas (https://www.proteinatlas.org/). **(H)** Representative NEDD4L immunohistochemical staining images with medium, low and not detected expression in melanoma patients. **(I)** The percent of immunohistochemical staining with medium, low and not detected expression in melanoma patients. **(J)** Kaplan-Meier analysis of NEDD4L expression in melanoma patients (n = 102, *P* = 0.0092). **(K)** Analysis of the interactions between NEDD4L and SP1 in A375 cells; by Immunoblotting. **(L)** Analysis of the purification protein GST-NEDD4L by Coomassie brilliant blue staining. **(M)** Analysis of the interactions between NEDD4L and SP1 by GST pull-down assay. **(N-O)** NEDD4L increases ubiquitination and degradation of SP1 in A375 cells; by immunoblotting.

To gain insight into the potential mechanisms of SP1 ubiquitination, we predicted the E3 ubiquitin ligase of SP1 by UbiBrowser, an integrated bioinformatics platform (http://ubibrowser.ncpsb.org); as shown in **Figure 4A**, more than a dozen E3 ubiquitin ligases were predicted as the candidates for SP1. Immunoblotting showed that only NEDD4L expression among top five candidates was activated by JP1 treatment in A375 cells** (Figure 4B, Figure S6A)**; the interaction region between NEDD4L and SP1 was identified as **Figure 4C**. The induction of JP1 on NEDD4L was further confirmed in both B16F10 cells allograft and metastatic tumor tissues by Immunoblotting **(Figure 4D-4E)** and IHC assay **(Figure 4F-4G)**. The correlations between NEDD4L and the prognosis of melanoma were then verified by database of The Human Protein Atlas; as shown in **Figure 4H-4I**, in 20 cases of melanoma tissue, NEDD4L is moderately expressed in 2 cases, low in 10 cases, and undetected in 8 cases; moreover, the patients with high expression of NEDD4L in melanoma tissues showed better overall survival than those with low expression ones **(Figure 4J)**. To verify the interactions between NEDD4L and SP1, we constructed high- and low-NEDD4L A375 cell lines by Flag-NEGG4L and si-NEDD4L transfections respectively **(Figure S6B)**; the colony formation and transwell assays were then conducted for these A375 cells. The results showed that high expression of NEDD4L significantly inhibited the proliferation and migration of A375 cells **(Figure S6C-S6E)**. The interactions between NEDD4L and SP1 were also confirmed by Co-IP assays in both A375 and MEWO cells **(Figure 4K, Figure S6F)**. Furthermore, GST pull-down assays further confirmed the interactions between NEDD4L and SP1 **(Figure 4L-4M)**. More importantly, NEDD4L mediated ubiquitination of SP1 was verified in both A375 and MEWO cells **(Figure 4N-4O, Figure S6G-S6H)**, respectively. The colony formation and transwell assays showed that increased proliferation and migration of cells due to reduced SP1 were neutralized by increased NEDD4L** (Figure S6C-S6E)**. These data indicate that NEDD4L mediates the degradation of SP1 by an ubiquitin-proteasome pathway.

### The K685 site of SP1 is required for its ubiquitination by NEDD4L

**Figure 5:**
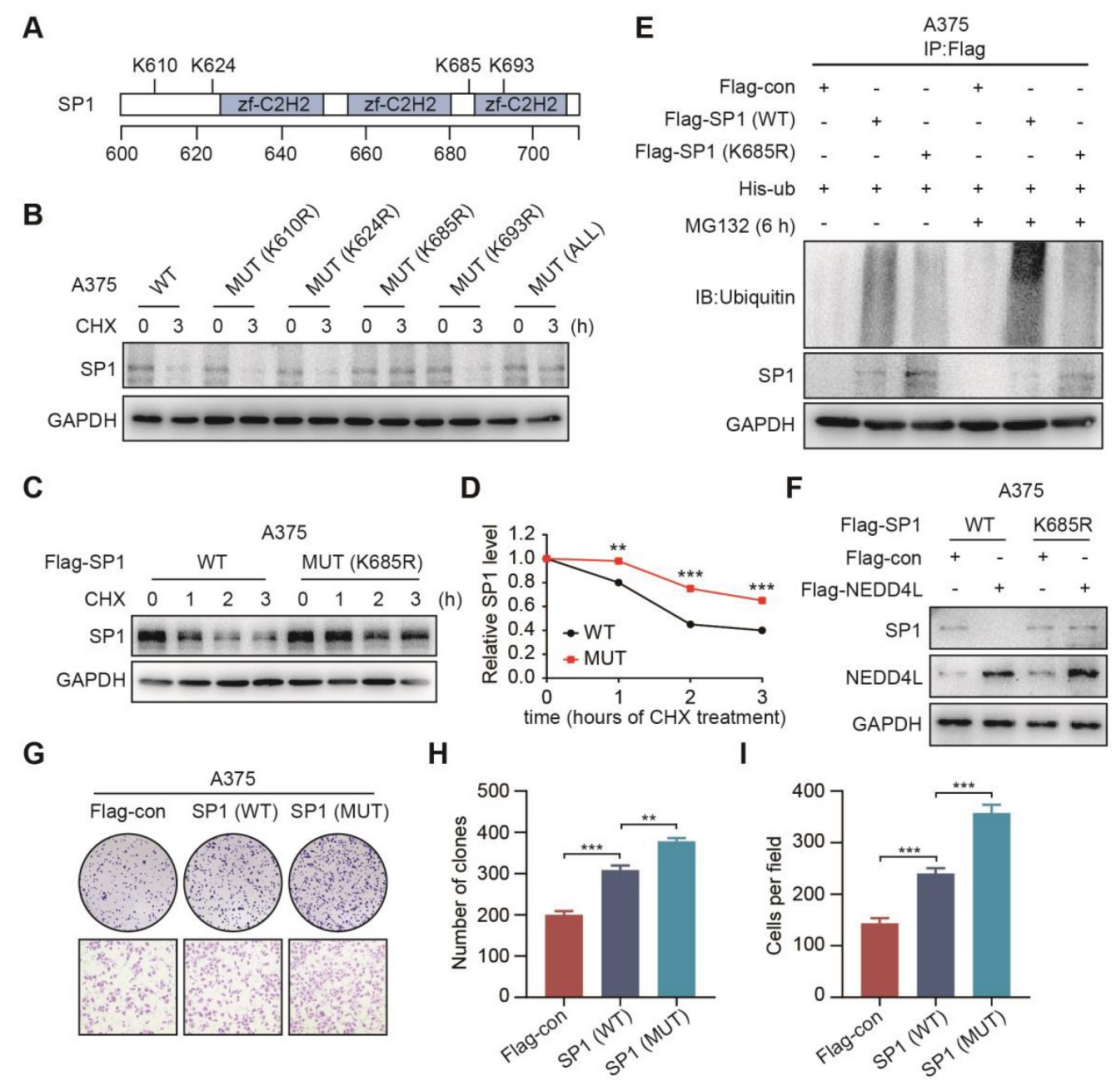
** The K685 site of SP1 is required for its ubiquitination by NEDD4L. (A)** Schematic representation of the potential ubiquitinated sites in SP1 protein. **(B)** The expressions of SP1 WT or mutants in A375 cells; by Immunoblotting. **(C-D)** The expressions **(C)** and time course levels **(D)** of Flag-SP1 (WT or K685R) in A375 cells; by Immunoblotting. n = 3; **P < 0.01, ***P < 0.001.** (E)** The ubiquitinations and expressions of Flag-SP1 (WT or K685R) in A375 cells; by Immunoblotting. **(F)** The interactions between Flag-NEDD4L and Flag-SP1 (WT or K685R) in A375 cells; by Immunoblotting.** (G-I)** The colony formation **(G-upper, H)** and transwell assays **(G-lower, I)** for A375 cells transfected Flag-SP1 (WT or K685R), respectively; n = 3. **P < 0.01; ***P < 0.001.

To find out the potential sites that were required for SP1 ubiquitination, we predicted four potential lysine sites (K610, K624, K685 and K693) in SP1 protein sequence (https://www.phosphosite.org/) **(Figure 5A)**; the four K sites was then mutated to arginine; and transfected SP1 wild type (WT) or the mutant (MUT) plasmids into A375 cells, respectively; and with or without treatment of CHX for 3 h. Immunoblotting showed that both the K685R mutant and all 4-site mutant plasmids transfected cells were resistant to CHX accelerated degradation of SP1 **(Figure 5B)**. To confirm this, we repeated to transfect WT or K685R mutant plasmids into A375 and MEWO cells and treated cells with CHX for 0, 1, 2, 3 h, respectively. It was shown that the SP1 expression in cells transfected K685R mutant plasmid was more stable than in those with WT plasmid** (Figure 5C-5D, Figure S7A-S7B)**. Importantly, the ubiquitination of SP1 was prevented in cells with SP1 (K685R) compared to the cells with SP1 (WT) **(Figure 5E)**. We further confirmed that the cells with SP1 (K685R) were resistant to NEDD4L-induced degradation of SP1 **(Figure 5F, Figure S7C)**. Both colony formation and transwell assays also showed that cells with SP1 (K685R) were enhanced proliferation and migration compared to the cells with SP1 WT **(Figure 5G-5I)**. These results suggest that the K685 site of SP1 is necessary for its ubiquitination by NEDD4L and linked cell proliferation and migration.

### JP1 stabilizes NEDD4L by promoting its phosphorylation

**Figure 6:**
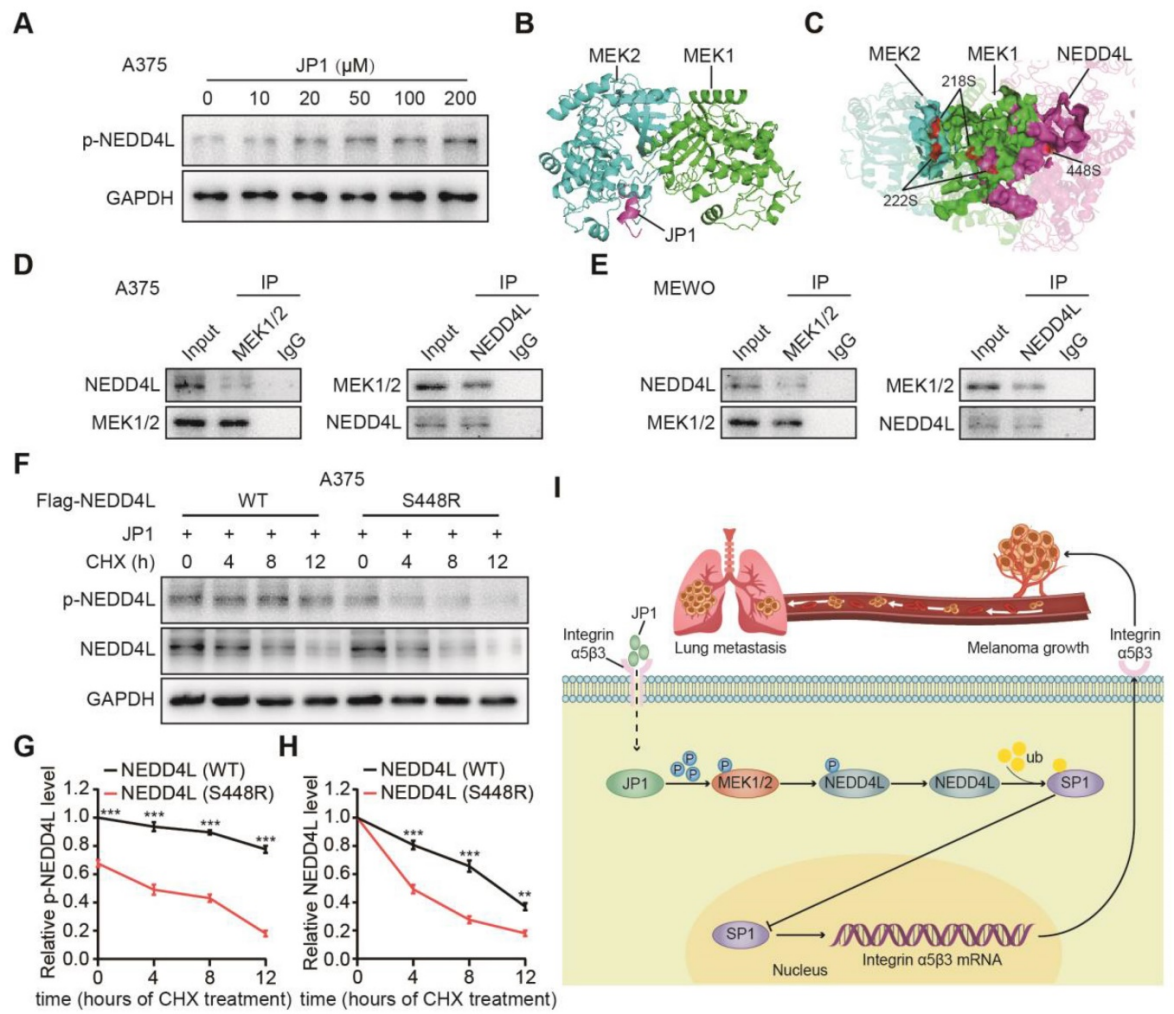
** JP1 stabilizes NEDD4L by promoting its phosphorylation. (A)** JP1 dose-dependently induced expressions of p-NEDD4L in A375 cells; by Immunoblotting.** (B)** The calculated protein-protein interaction model between JP1 and MEK1/2.** (C)** The calculated protein-protein inactions among JP1-MEK1/2-NEDD4L. The 218/222 sites of MEK1/2 and the 448 sites of NEDD4L were located in the hotspot regions of interaction. **(D-E)** Analysis of the interactions between MEK1/2 and NEDD4L in A375 **(D)** and MEWO **(E)** cells; by Immunoblotting. **(F-H) (F)** The effects of JP1 on the stability of NEDD4L (WT or S448R) in A375 cells; by Immunoblotting. The time-course intensities of the p-NEDD4L **(G)** and NEDD4L **(H)** in A375 cells after transfection of NEDD4L (WT or S448R); n = 3. **P < 0.01, *** P < 0.001.** (I)** The graphic illustration of molecular mechanism of JP1 suppressing melanoma growth and metastasis.

How does JP1 activate NEDD4L in melanoma cells? Several studies show that NEDD4L stabilizes its expression in tumors mainly through its self-phosphorylation 26, 27. To determine p-NEDD4L by JP1, we analyzed phosphorylated expression levels of NEDD4L (p-NEDD4L). The results showed that JP1 enhanced the expression of p-NEDD4L **(Figure 6A, Figure S8A)**. To further identify the potential mechanisms that JP1 activates NEDD4L, we completed molecular docking analysis. As a result, JP1- MEK1/2 and JP1-MEK1/2-NEDD4L interaction complex were identified **(Figure 6B-6C, Figure S8B-S8C, Table S1-S3)**. Co-IP assays confirmed the interactions between MEK1/2 and NEDD4L in both A375 and MEWO cells **(Figure 6D-6E)**. In addition, as shown in **Figure 6C**, both the 218/222 sites of MEK1/2 and the 448 site of NEDD4L were included in the protein-protein interaction regions, i.e. ATP binding pocket. Hence, the 448 site of NEDD4L might be modified by phosphorylation. To confirm this, we constructed both the wild and 448-site mutant plasmids of NEDD4L and then transfected these into A375 and MEWO cells, respectively. With the extension of CHX exposure time, the expression level of p-NEDD4L protein in cells transfected with NEDD4L wild plasmid was significantly higher than that in cells transfected with NEDD4L mutant plasmid **(Figure 6F-6G, Figure S8D-S8E).** More importantly, the protein expression level of NEDD4L was also showed a consistent trend with p-NEDD4L expression **(Figure 6F and 6H, Figure S8D and S8F)**. Suggesting that JP1 treatment increased phosphorylation of MEK1/2 and further activated p-NEDD4L. The phosphorylation of NEDD4L increased its stability.

To determine the stability of JP1 as a therapeutic agent, we completed pharmacokinetic assays. Data showed that the half-life of JP1 by intraperitoneal injection in rats was about 1.317 h **(Figure S9A-S9B)**. To improve the stability of JP1 peptide *in vivo*, we designed and replaced the linear JP1 with circular design, PEG and palmitic acid modifications, respectively. Unfortunately, the results showed that only the linear JP1 polypeptide indicated obvious anticancer effect on melanoma **(Figure S10A-S10F)**.

In summary, we developed a novel therapeutic JP1 peptide on melanoma. JP1 was targeted to and entered melanoma cells with high expression of integrin αvβ3 by RGD, and then interacted with MEK1/2 to activate E3 ubiquitination enzyme NEDD4L, which accelerated the degradation of SP1 and ultimately played a transcriptional inhibitory role on integrin αvβ3. JP1 exerted tumor inhibition effects through an integrin α5β3-mediated close-loop mechanism **(Figure 6I)**.

## Discussion

In the present study, we reported a novel JP1 therapeutic peptide for treatment of metastatic melanoma. JP1 was designed a pre-phosphorylated seven amino acid fragment and with RGD linker to target integrin αvβ3. The inhibitory roles of JP1 on proliferation and metastasis of melanoma were determined through MEK1/2-NEDD4L-SP1-Integrin αvβ3 signaling. In addition, JP1 in combination with DTIC indicated synergistic and detoxifying effects on melanoma. These evidences suggest that JP1 has the potential to be a novel drug on melanoma.

The advantages of JP1 as a therapeutic peptide of melanoma may include but not limit as: (1) Extensibility of use. JP1 targeted and down-regulated integrin αvβ3 which was extensively overexpressed in most cancer cells, such as melanoma, gastric cancer, pancreatic cancer, hepatocarcinoma, breast and lung cancer, etc. Therefore, JP1 may be suitable for kinds of cancers; (2) For multiple anticancer combination therapies include dacarbazine since JP1 has shown anticancer effects in both immune normal/deficiency mouse models. The synergistic anticancer effects in JP1 combination with immunotherapy is promising; (3) JP1 was an endogenous molecule that without immunogenicity; the therapeutic role of JP1 was due to reverse the disordered signal pathways in cancer cells; and (4) the biological half-life of JP1 was longer in cells than that in blood.

Although several cancer therapies except for surgery are available for patients, the drug induced toxicities and the secondary resistance remain big challenges worldwide 28, 29. That's mostly because we haven't really figured out the characteristics how normal cells become cancer cells 30. We know that all cancer cells have disorders in their signaling networks but we don't know how to correct them 31, 32. JWA is an evolutionary-conservative gene whose expression levels are reduced in most kinds of cancer cells, resulting in the disorder of cellular signaling networks 20, 33. JP1 properly replenishes the lost JWA protein in cancer cells and corrects the integrin αvβ3 related signal network, so that some phenotypes of cancer cells could be returned to near normal level. The obvious tumor-suppressive effect and biosafety of JP1 provided a novel strategy for reducing the toxicity of DTIC in treatment of melanoma.

The therapeutic peptides have gained much interest as anticancer drug development because they are recognized for being highly selective and efficacious and, at the same time, relatively safe and well tolerated 11, 34. However, the instability of peptide drugs is still needed to be improved in drug development 35. Some progress has been made in the use of subcutaneous slow-release pump and the study of oral polypeptide drugs, which are expected to be used in the clinical treatment of polypeptide drugs in the future 36, 37. In addition, the combination of peptide drugs and other drugs or therapies may become a feasible way for cancer therapy 38, 39.

Finally, it should be pointed out although JP1 peptide plays a significant role in the treatment of melanoma by targeting integrin α5β3; the therapeutic effect of JP1 may quite different among individuals even with same cancer, which may be caused by tumor heterogeneity 40. In some heterogeneous tumor cells, it may not be integrin α5β3 but other molecules overexpressed, such as MMP2 or VEGFR. A joint combination of the appropriate peptides with different targeted molecules might be achieved the precise treatment of heterogeneous tumors.

## Materials and Methods

### Peptides synthesis

All peptides were synthesized by GL Biochem (Shanghai) Ltd. and Hybio Pharmaceutical Co., Ltd. (Shenzhen, China; JP1 was synthesized under standard GMP condition, and special used for monkey toxicity test), respectively; with purities > 98% and confirmed by HPLC-MS analysis. The JP1 was synthesized as pre-phosphorylated at serine and a routine modifications of acetylation and amidation at the N- and C-termini, respectively. The freeze-dried peptide powder was always stored at -20 °C.

### Screening strategies for the anti-tumor peptides using mouse tumor models

Human melanoma cells (A375, 5 × 10^6^ diluted in PBS) were injected subcutaneously into the flanks of four to five-week-old female nude mouse (SLAC Laboratory Animal Center, Shanghai, China). Tumor dimensions were measured with electronic calipers. When the tumor volume reached about 100 mm^3^ in average (the tumor volume was calculated according to the formula: V = 0.5 × L × W^2^, where L is the longer dimension, and W is the shorter one), the mouse were randomly divided into different groups (n = 6 per group), and treated with different peptides, which was administered by intratumor injection. The body weights and tumor volumes were measured at the indicated time points.

### Synthesis of ^18^F-NFP-JP1 conjugates

All reagents were of analytical grade and were purchased from commercial suppliers and used without further purification. No-carrier-added ^18^F^-^ was produced by a GE Healthcare cyclotron (Key laboratory of Nuclear Medicine, Ministry of Health). Ethanol (5 ml) and deionized water (15 ml) were applied to treat the C_18_ Sep-Pak cartridges before use. A high-performance liquid chromatography (HPLC) system with a Waters 2998 photodiode array detector and a pretreated C_18_ HPLC column (5 μm, 250 × 19 mm, Waters Xbridge) were used for peptide purification. ^18^F-NFP was produced according to a previously reported protocol. Briefly, about 150 MBq of ^18^F^-^ was added into 100 μl of K222 compound buffer (150 mg K222 dissolved in 1 ml of acetonitrile containing 0.3 mg KCO_3_), and the mixture was heated at 115 °C for 15 min. During the reaction, 600 μl of acetonitrile was added, and a low stream of nitrogen was applied to dry the compound. Then, 7 mg of NFP dissolved in 400 μl of acetonitrile was added at 115 °C for another 10 min. After cooling, 50 μl of TBAH (tetrabutylammonium hydroxide) was added, followed by 100 μl of acetonitrile, and the mixture was dried under a nitrogen stream. A total of 40 mg of BC dissolved in 400 μl acetonitrile was added for another more 10 min, and 200 μl of acetonitrile containing 50% acetic acid was added for 5 min at 117 °C. The crude ^18^F-labeled NFP was purified by semi-preparative HPLC, and desired fractions were collected and concentrated to a powder at a > 85% yield. Then, 0.5 mg JP1 in 200 μl of DMSO and 20 μl of N, N-diisopropylethylamine was added to the ^18^F-NFP powder at 37 °C for 20 min. Semi-preparative HPLC was used to purify the products. The collected fractions were diluted in 15 ml water and passed through a C_18_ Sep-Pak cartridge, and the cartridge was washed with 0.3 ml of ethanol hydrochloride to obtain the purified ^18^F-NFP-JP1. Its retention time was 18.7 min.

### MicroPET imaging and blocking experiments

An Inveon microPET scanner (Siemens Medical Solution, Germany) was applied for mouse during PET imaging. Female BALB/c nude mouse (5-6 weeks-old) bearing B16F10 tumors in their right front flanks were anesthetized with 2% isoflurane, placed in the prone position, and immobilized in the scanner. Subsequently, ^18^F-NFP-JP1 (3.6 MBq in 100 μl of saline) was intravenously injected into the mouse. Static PET images were acquired for 10 min at 0.5, 1, and 2 h after injection. For the blocking experiments, Ctrl-peptide (150 mg/kg) was injected via the tail veins 30 min prior to ^18^F-NFP-JP1 administration. Then, 10-min static PET scans were performed 30 min after injection.

Human melanoma cells (A375, 5 × 10^6^ diluted in PBS) were injected subcutaneously into the flanks of four to five-week-old nude female nude mouse (SLAC Laboratory Animal Center, Shanghai, China). When the tumor volume reached 100 mm^3^, the mouse were randomly divided into three groups (n = 6 per group), and treated with Ctrl-R, JP1 or Ctrl-R + JP1 (Ctrl-R with 150 mg/kg was intraperitoneal injected at 30 min prior to JP1 administration), which was administered intraperitoneally. The body weights and tumor volumes were measured at the indicated times.

### Biodistribution studies

Female BALB/c nude mouse bearing B16F10 tumors were dosed with 1.8 MBq of ^18^F-NFP-JP1 via the tail vein and were sacrificed at 0.5, 1, or 2 h post-injection. All major organs, as well as the blood and tumors, were collected and weighed, and the radioactivity of each sample was analyzed with a γ-counter. In addition, three mouse were intravenously injected with Ctrl-R (150 mg/kg body weight) 0.5 h before ^18^F-NFP-JP1 administration. After 30 min, these mouse were sacrificed, and the radioactivity in each sample was determined. The data were presented as a percent of the injected dose per gram of tissue (%ID/g).

### Melanoma growth and metastasis model

C57BL/6 male mouse (5-6 weeks old) and BALB/c male mouse (4-5 weeks old) were purchased from SLAC Laboratory Animal Center (Shanghai, China) and maintained in SPF facilities. B16F10 cells (5×10^5^) were subcutaneously injected into the right axilla of C57BL/6 male mouse (MEWO cells (5×10^5^) for BALB/c male mouse). When the tumor volume reached 100 mm^3^ (the tumor volume was calculated according to the formula: V = 0.5 × L × W^2^), the mouse was randomly divided into two groups (n = 6 per group), and treated with Ctrl-R or JP1, which was administered intraperitoneal injection. The body weights and tumor volumes were measured at the indicated times. At the end of the experiment, the mouse were sacrificed, and the tumors were weighed and imaged and then frozen for further analyses.

The C57BL/6 mouse were injected with 2 × 10^5^ B16F10 cells in 0.1 ml of PBS through tail vein before the melanoma metastasis model began. Then the mouse were randomly divided into two groups (n = 6 per group) and treated with Ctrl-R or JP1, which was administered intraperitoneal injection. After 21 days, the mouse were sacrificed, and the lung metastatic nodules were taken for frozen in -80 °C freezer, and for further analysis. The number of metastatic foci and the area of lung metastases were examined by histological examination of the indicated lung tissue sections. If survival analysis was performed, the natural death time of the mouse were recorded.

To simulate the occurrence of clinical tumor metastasis, a melanoma active metastasis model was performed. B16F10 cells (5 × 10^5^) were subcutaneously injected into the right axilla of C57BL/6 mouse. When the tumor volume reached 100 mm^3^, the mouse were randomly divided into two groups, and treated with Ctrl-R or JP1 (6 mouse of Ctrl-peptide, 12 mouse of JP1). When the tumor volume reached about 2000 mm^3^, the tumor tissue was taken out by aseptic surgery (The JP1 component was divided into two groups, one with JP1 intervention and the other with PBS intervention). On the 40^th^ day after the tumor-bearing day, the mouse were sacrificed and the incidence of lung metastasis were counted.

### Cynomolgus monkey toxicity test

The test was completed by Suzhou Xishan Zhongke Drugs Research and Development Co., Ltd. (Xishan, Suzhou, China). The study proposal was approved by the Institute Animal Care and Use Committee (IACUC, ID19012912). Cynomolgus monkeys (3-4 years old) were randomly divided into three groups (n = 4 per group, half male and female); and the monkeys were treated with solvent control (Mannitol in saline, 75 mg/kg/d), JP1 (45 mg/kg/day) and JP1 (150 mg/kg/day) for 14 days. The experiment was ended on 15^th^ day and the animals were euthanasia. The relevant clinical parameters were determined on 7^th^ and 13^th^ day, respectively. The observed parameters were including clinical symptoms, weight, food intake, body temperature, ophthalmology, electrocardiogram, blood routine examination, blood biochemistry, blood coagulation, urinalysis, immunotoxicity.

### Cell lines and cell culture

Human melanoma cell lines (A375, MEWO) and mouse melanoma cell line (B16F10) were purchased from ATCC (MD, USA). All the cell lines were cultured in DMEM and supplemented with100 μg/ml streptomycin, 100 U/ml penicillin and 10% fetal bovine serum in an incubator with 5% CO_2_ at 37 °C.

### Plasmids and siRNA transfection

The commercialized SP1 and NEDD4L plasmids were purchased, the siNEDD4L were designed and synthesized, all mutation plasmids (610, 624, 685, 693 sites of SP1 and 448 site of NEDD4L) were synthesized from Shanghai Genechem Co., Ltd. The DNA plasmids or siRNA were transfected into cells with Lipofectamine 3000 (Invitrogen, Grand Island, NY, USA) according to the manufacturer's instructions.

### Immunoblotting

Immunoblotting was conducted as reported previously. Briefly, cell samples were lysed in lysis buffer (50 mM Tris, pH 7.4; 150 mM NaCl; 1% NP-40; 0.5% sodium deoxycholate; 0.1% SDS; and the protease inhibitor, 1 mM PMSF), and tissue samples were prepared in tissue protein extraction reagent (Thermo Fisher Scientific). Protein (40 μg) was processed for the analysis. The antibodies used for the analysis were as follows: anti- Integrin α5 (1:200, Santa Cruz Biotechnology), anti-Integrin β3 (1:1000, Cell Signaling Technology), anti-SP1 (1:1000, Proteintech), anti-p-MEK1/2 (Ser218/222, 1:1000, Cell Signaling Technology), anti-MEK1/2 (1:1000, Cell Signaling Technology), anti-p-NEDD4L (Ser448, 1:1000, Abcam), anti-NEDD4L (1:1000, Proteintech), anti-ubiquitin (1:500, Santa Cruz Biotechnology), anti-GAPDH (1:1000, Beyotime).

### Immunoprecipitation and ubiquitination assay

Immunoprecipitation procedure was conducted as described previously. In short, cells were treated as indicated and were lysed in TNE buffer (50 mM Tris-HCl, pH 7.4, 150 mM NaCl, 1% NP40) containing protease inhibitors (Sigma). The cell lysates, cleared by centrifuging at 12,000 × g for 15 min, were incubated with the indicated antibodies at 4 °C overnight. Next, lysates were incubated with protein A/G agarose beads (Santa Cruz) for 2 h at 4 °C. The beads were then collected by centrifugation, washed five times with TNE buffer, suspended in 2 × SDS loading buffer, and proteins were detected by Immunoblotting. For ubiquitination assay, cells were treated with MG132 (10 μM) for another 6 h before lysed.

### Protein purification and pull-down assay

The expression vectors with either GST-labeled NEDD4L or His-labeled SP1 were constructed by Shanghai Genechem Co., Ltd. GST Spin Purification Kit (16107) and GST Protein Interaction Pull-Down Kit (21516) were purchased from Thermo.

Protein purification: briefly, centrifuging column at 700 × g for 2 min to remove storage buffer and equilibrate column with two resin-bed volumes of equilibration buffer. Add the prepared protein extract to the column and allow it to enter the resin bed. Then centrifuging column at 700 × g for 2 min and collect the flow-through in a centrifuge tube. Washing resin with two resin-bed volumes of equilibration buffer. Centrifuging at 700 × g for 2 min and collect fraction in a centrifuge tube. Eluting GST-tagged protein from the resin by adding one resin-bed volume of elution buffer. Protein pull-down assay: briefly, immobilizing the obtained GST-NEDD4L protein on the glutathione agarose according to the instructions. Adding in prepared prey protein sample and incubated at 4 °C for at least 1 h. Centrifuging at 1250 × g for 30 s to 1 min. Adding in 400µL of wash solution and repeat washing for a total of five washes. Eluting with glutathione elution buffer and collecting eluent for analysis.

### Intracellular distribution of FITC-JP1

The FITC-JP1 was synthesized by GL Biochem (Shanghai) Ltd with purities > 98% confirmed by HPLC. The mouse melanoma B16F10 cells were treated with different doses of FITC-JP1 for 3 h, followed by washing with PBST 3 times, then were fixed with methanol for 30 min. Subsequently, the nuclei were counterstained with DAPI (Beyotime, Shanghai, China) for 15 min. Images of the cells were acquired with a Zeiss LSM 700 confocal microscope system (Carl Zeiss Jena, Oberkochen, Germany).

### Proliferation and transwell migration assays

For the Proliferation assay, 500 melanoma cells were seeded in 6-well plates and incubated at 37 °C. After two weeks, cells were fixed and stained with crystal violet (Beyotime, Shanghai, China) for 30 min.

The migration assays were conducted using Transwell™ filter, a modified two-chamber plate with a pore size of 8 μm. The treated melanoma cells were seeded in serum-free medium in the upper chamber and the medium with 10% FBS was added to the lower compartment. After 12 h of incubation at 37 °C, the melanoma cells in the upper chamber were carefully removed using a cotton swab, and the cells in the lower compartment were stained with crystal violet (Beyotime, Shanghai, China) for 30 min.

### Protein structure analysis

The iterative threading assembly refinement (I-TASSER) server is an integrated platform for automated protein structure prediction based on sequence-to-structure paradigm. Three dimension structures of MEK1/2, JP1, and NEDD4L were then predicted using the I-TASSER server. The largest possible binding pocket of these proteins, i.e., MEK1/2 and NEDD4L, was then predicted by Discovery Studio 3.0, respectively. These predicted pockets were utilized to construct an initial coarse model of the MEK1-MEK2 complex, MEK1/2-JP1 complex, and JP1-MEK1/2-NEDD4L complex. Then, complexes were refined by Rosetta software (RosettaDock and FelxPepDock module). The optimization model for complex was then obtained based on energy scores. Binding sites/regions between proteins in complex were then obtained by RING (residue interaction network generator). High-quality 3-D images of structures were drawn by PyMol.

### Pharmacokinetic analysis

A preliminary pharmacokinetic analysis of JP1 in Sprague-Dawley rats (SD rats) was completed by Shanghai Medicilon Inc. Two male SD rats (6-8 weeks old) were injected intraperitoneally with 100 mg/kg of JP1, and the blood was collected after JP1 injection for 0.083, 0.25, 0.5, 1, 2, 4, 8, 24 h. The concentration of JP1 in the blood was measured by LC-MS/MS system. The drug half-life (t_1/2_) is calculated by the formula t_1/2_ = 0.693/k, where k is the elimination rate constant (k = (Inc_1_-Inc_2_)/(t_2_-t_1_)). In this study, the mean blood concentration in two SD rats were 20452.06 ng/ml after JP1 exposure for 0.25 h, and the mean blood concentration was decreased to 13789.69 ng/ml after 1 h, then k = (In20452.06-In13789.69)/(1-0.25) = 0.526. Therefore, the calculated half-life of JP1 with intraperitoneal injection in rats was 1.317 h.

### Statistics analysis

Data were analyzed using GraphPad Prism 8. Statistical significance (*P* < 0.05) between the means of two groups was determined using the Dunnett's t-test. The difference in survival between two groups were calculated using the log-rank test. *P* < 0.05 was considered statistically significant.

## Supplementary Material

Supplementary figures and tables.

## Abbreviations

CHX: Cycloheximide
DTIC: Dacarbazine
FITC: Fluorescein isothiocyanate
HPLC: High Performance Liquid Chromatography
JP1: JWA peptide 1
MEK1/2: MAP kinase kinase1/2
micro-PET: Micro positron emission tomography
NEDD4L: Neural precursor cell expressed, developmentally down-regulated 4-like
