# Supplementary material for: JP1 suppresses proliferation and metastasis of melanoma through MEK1/2 mediated NEDD4L-SP1-Integrin αvβ3 signaling
Source: Theranostics. 2020 Jul 1;10(18):8036–50. doi: 10.7150/thno.45843 (PMC7381750; doi:10.7150/thno.45843)
Supplement: Supplementary file 1 — Supplementary figures and tables. [file thnov10p8036s1.pdf]

JP1 suppresses proliferation and metastasis of melanoma through MEK1/2 mediated NEDD4L-SP1-Integrin αvβ3 signaling

Jiahua Cui[1,2§], Chuanjun Shu[3§], Jin Xu[1,2], Dongyin Chen[4], Jin Li[5], Kun Ding[1,2], Minjuan Chen[1,2], Aiping Li[1,2], Jingdong He[5], Yongqian Shu[2,6], Liuqing Yang[7], Ruiwen Zhang[8], Jianwei Zhou[1,2*]

Supplementary figures

Figure S1. Screening strategies and targeting verification for anticancer peptides

Figure S2. JP1 inhibits the growth and metastasis of melanoma *in vivo*

Figure S3. JP1 reduces the dosage and toxicity in combination with DTIC

Figure S4. Cynomolgus monkeys toxicity test

Figure S5. JP1 mediated degradation of SP1 by the ubiquitin-proteasome pathway

Figure S6. E3 ubiquitin ligase NEDD4L inhibits melanoma cell proliferation and migration through ubiquitination of SP1

Figure S7. Mutation of K685 site in SP1 prevents its ubiquitination

Figure S8. JP1 stabilizes NEDD4L protein expression by activating p-NEDD4L

Figure S9. Pharmacokinetic of JP1 in SD rats

Figure S10. JP1-based modifications of peptides do not show inhibition of the malignant phenotype of melanoma *in vivo*

Supplementary tables

Table S1. The interaction sites of MEK1 and MEK2

Table S2. The interaction sites of JP1 and MEK2

Table S3. The interaction sites of MEK1 and NEDD4L

Supplementary database files: Cynomolgus monkey toxicity test

Table S4. Body weight

Table S5. Blood routine examination

Table S6. Blood biochemistry

Table S7. Blood coagulation

Table S8. Urinalysis

Table S9. Immunotoxicity

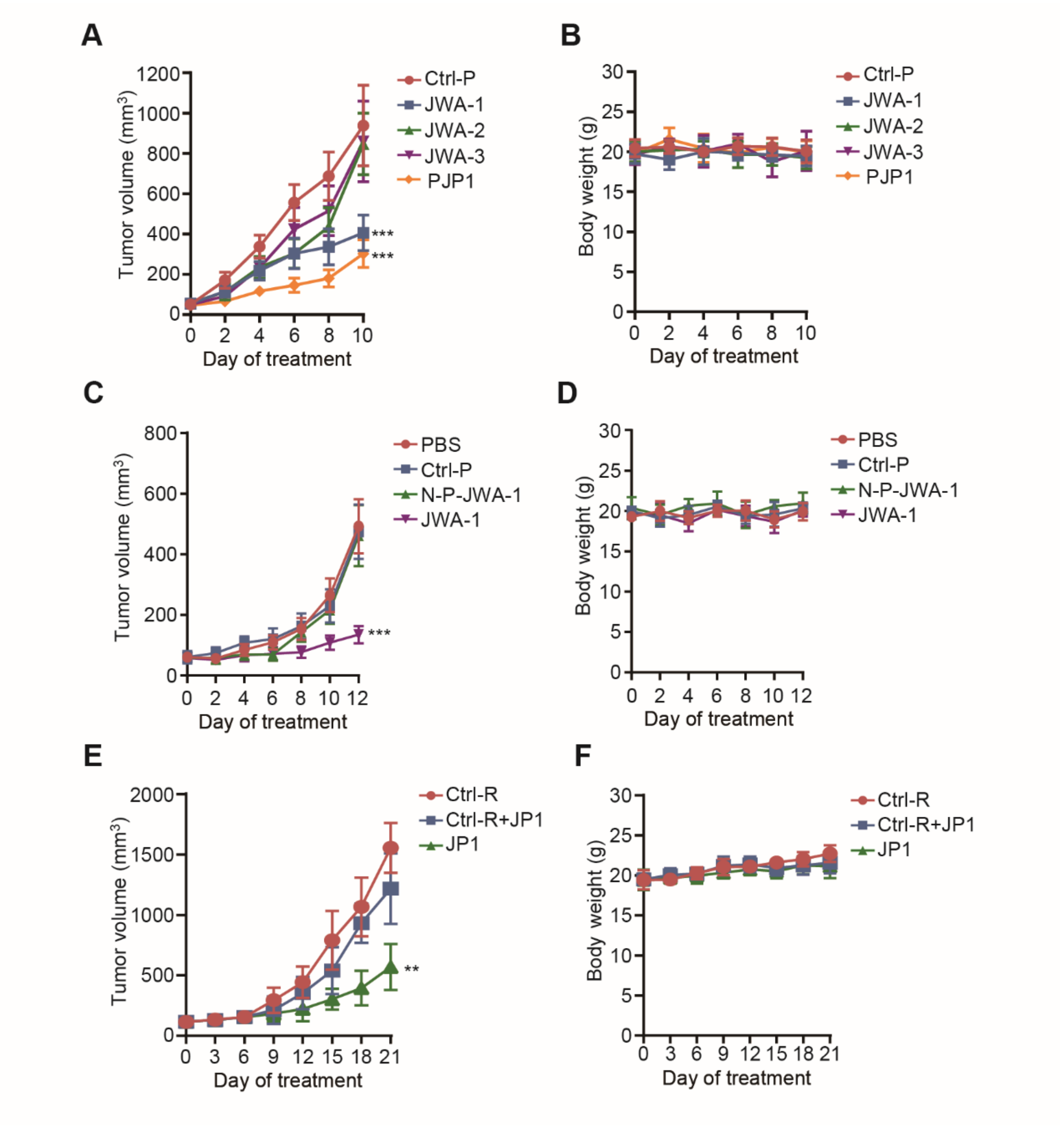

Figure S1. Screening strategies and targeting verification for anticancer peptides

(A, B) The subcutaneous tumors volume measurements **(A)** and body weights **(B)** at the indicated time points in different length peptides treatment group. **(C, D)** The subcutaneous tumors volume measurements **(C)** and body weights **(D)** at the indicated time points in phosphorylated and non-phosphorylated modification peptides treatment group. **(E, F)** The subcutaneous tumors volume measurements **(E)** and body weights **(F)** at the indicated time points in Ctrl-R, JP1 and Ctrl-R+JP1 treatment group. $^{**}P < 0.01$, $^{***}P < 0.001$.

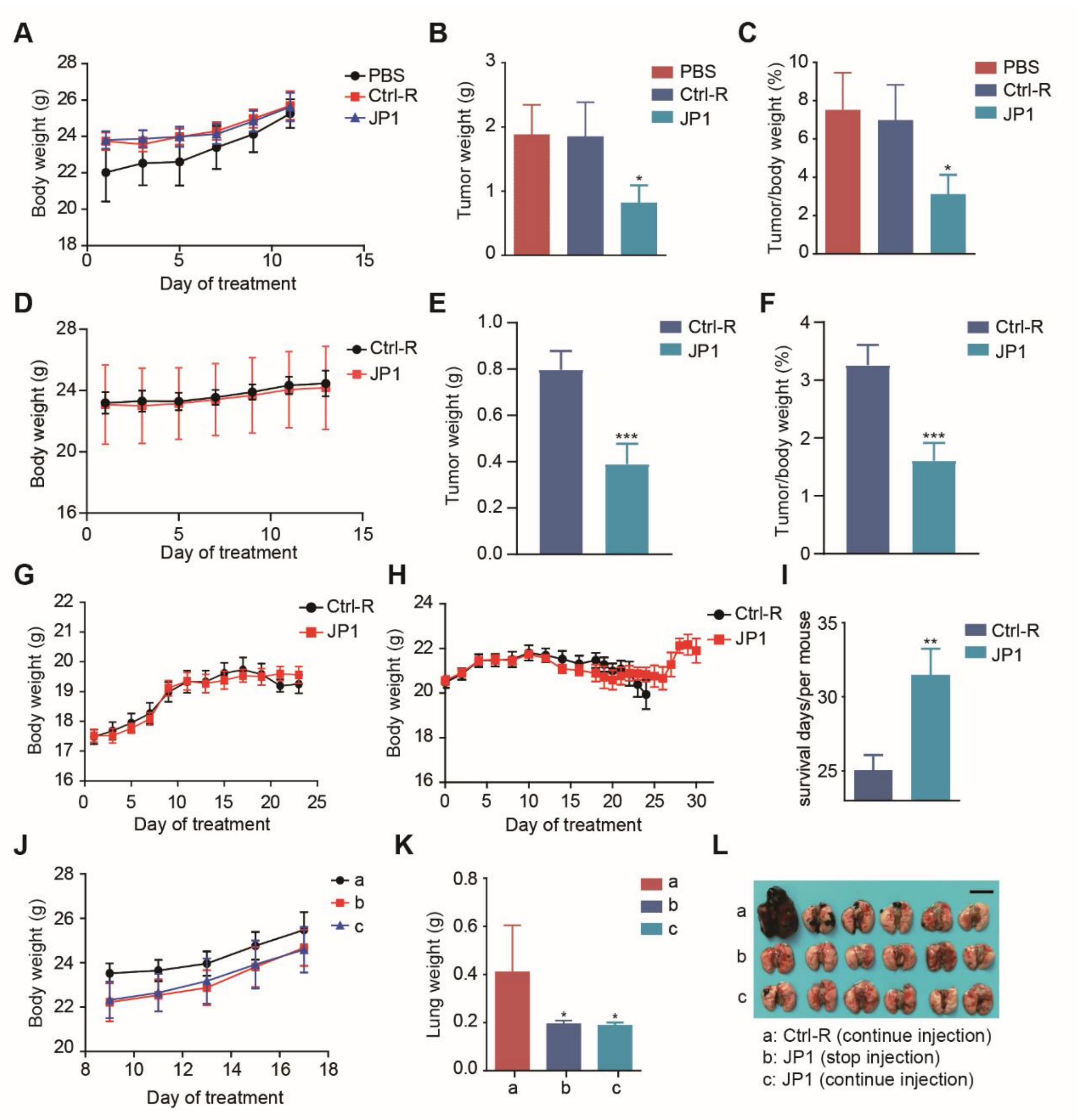

Figure S2. JP1 inhibits the growth and metastasis of melanoma in *vivo*

(A-C) The body weights **(A)**, tumor weights **(B)** and tumor/body weight ratio **(C)** of PBS, Ctrl-R and JP1 treated mouse in melanoma tumor-bearing model (B16F10 cells injection). **P* < 0.05. **(D-F)** The body weights **(D)**, tumor weights **(E)** and tumor/body weight ratio **(F)** of Ctrl-R and JP1 treated mouse in melanoma tumor-bearing model (MEWO cells injection). **P* < 0.05. **(G)** The body weights of Ctrl-R and JP1 treated mouse in melanoma passive transfer model. **(H, I)** The body weights **(H)** and survival days/per mouse **(I)** of Ctrl-R and JP1 treated mouse in melanoma survival model. **P* < 0.05. **(J-L)** The body weights **(J)** and lung weights **(K)** of Ctrl-R and JP1 treated mouse in melanoma active metastasis model. **(L)** Images of six lung metastases for each of the indicated treatment groups. Scale bar, 1 cm. **P* < 0.05.

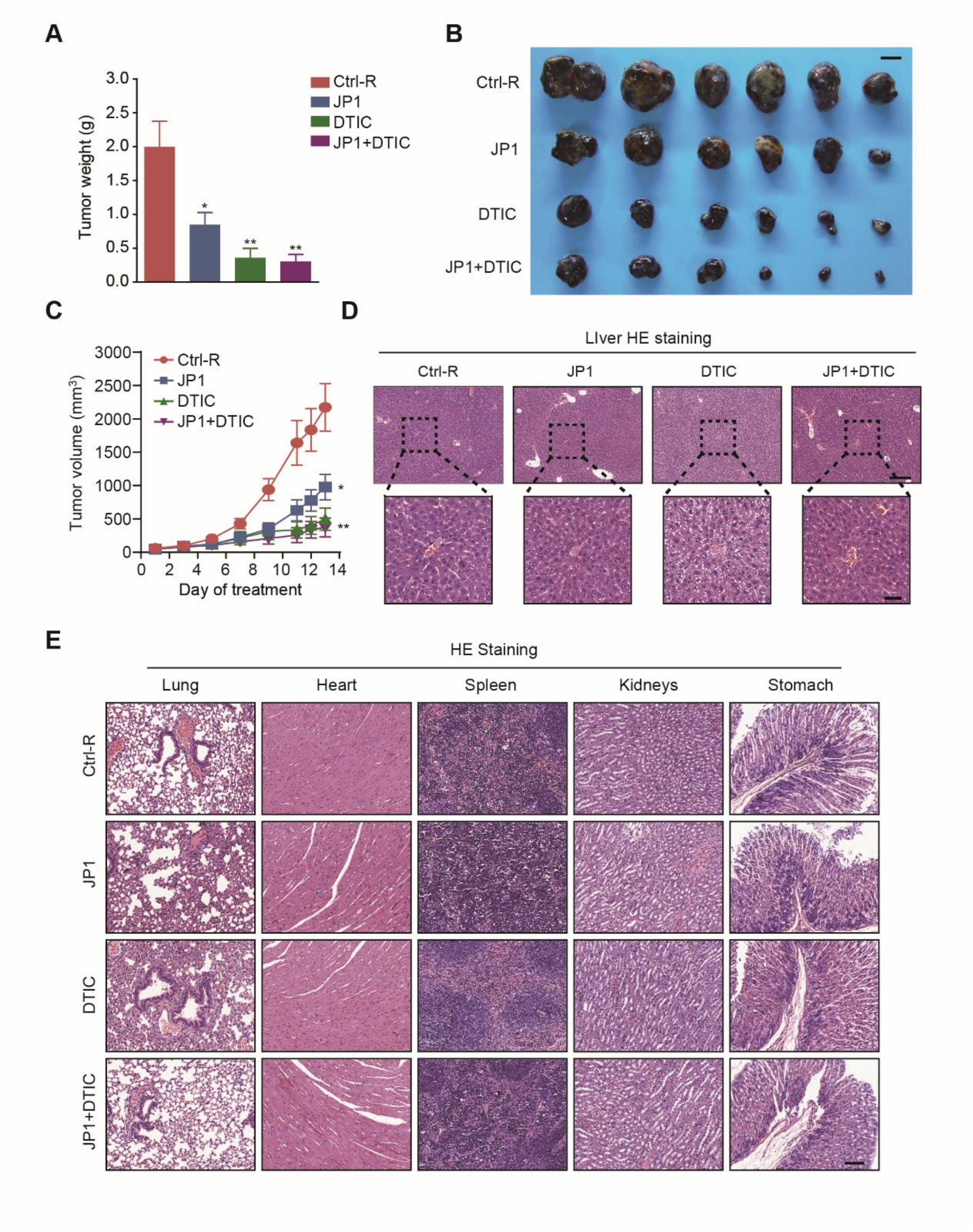

Figure S3. JP1 reduces the dosage and toxicity in combination with DTIC

(A) The tumor weights of 50 mg/kg Ctrl-R, 50 mg/kg JP1, 80 mg/kg DTIC and 50 mg/kg JP1 + 40 mg/kg DTIC treated mouse in melanoma tumor-bearing model. **P* < 0.05, ***P* < 0.01. **(B)** Images of six subcutaneous tumors for each of the indicated treatment groups. Scale bar, 1 cm. **(C)** The subcutaneous tumors volume measurements at the indicated time points in different

treatments. *P < 0.05, **P < 0.01. **(D)** Representative images of liver H&E staining after indicated treatments. Scale bar, 200 μm and 50 μm, respectively. **(E)** Representative lung, heart, spleen, kidney and stomach HE staining images in different treatment groups were shown. Scale bar, 100 μm.

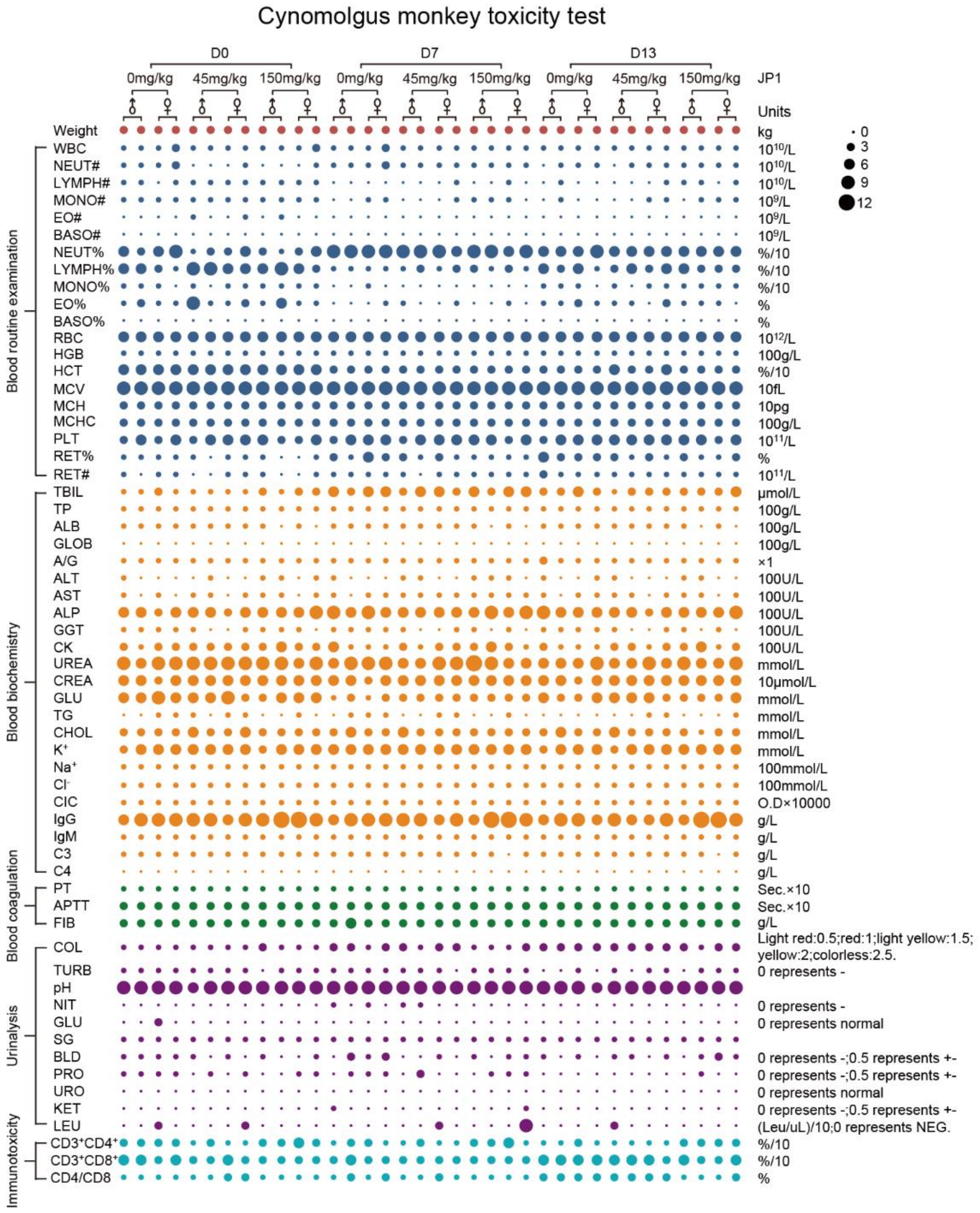

Figure S4. Cynomolgus monkeys toxicity test

The representative results from Cynomolgus monkeys toxicity test after JP1 treatments. Data shown the relative parameters detected on day 0, 7 and 13 and indicated treatments, respectively. The colour of dots indicates the parameters of sub-group and the diameter of dots represents the intensities of the parameters. Non-significant JP1-treatment related changes were observed, NOAEL = 150 mg/kg.

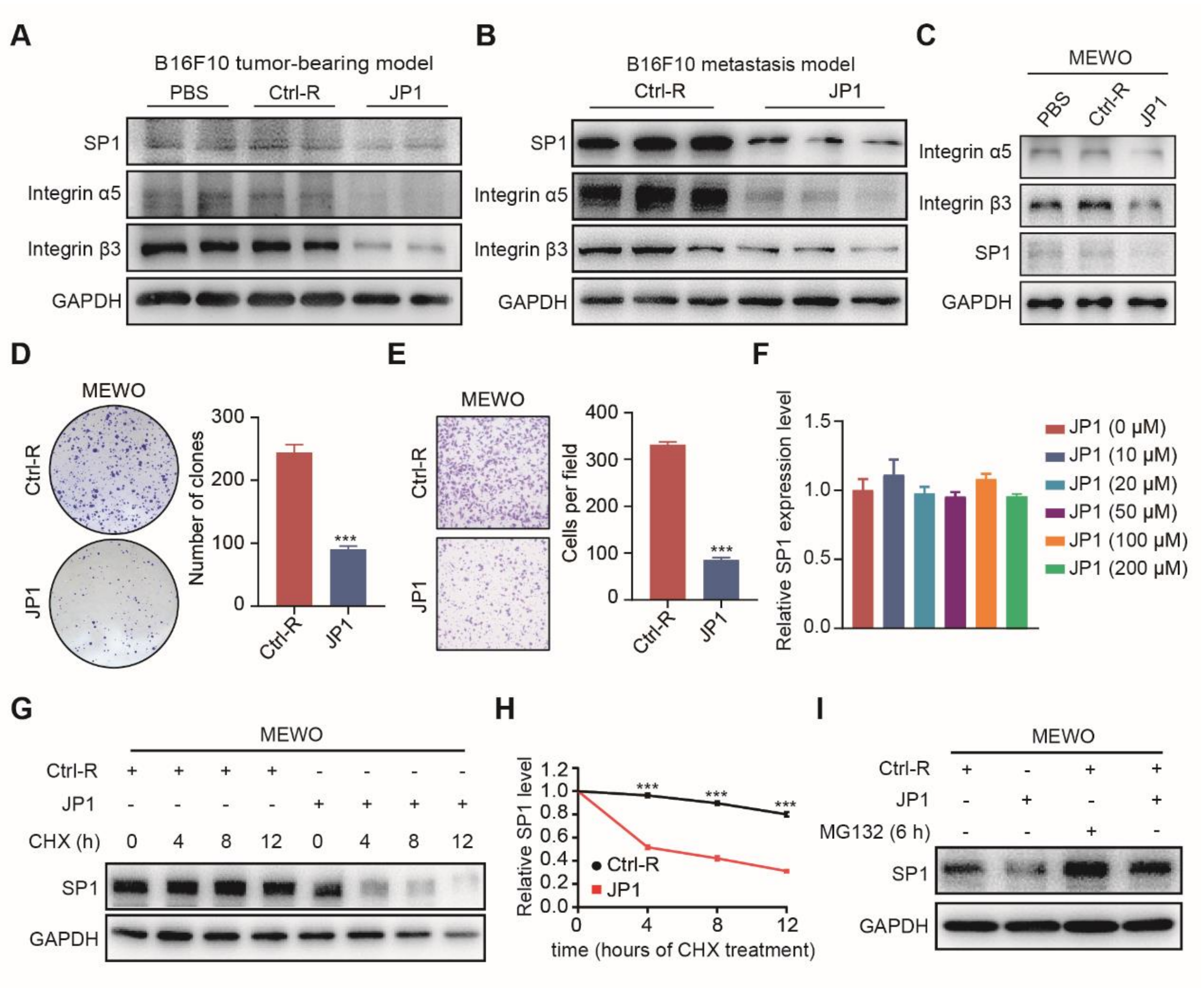

Figure S5. JP1 mediated degradation of SP1 by the ubiquitin-proteasome pathway

(A, B) In melanoma tumor-bearing model **(A)** and melanoma metastasis model **(B),** the expression levels of SP1, Integrin α5, and Integrin β3 in PBS, Ctrl-R and JP1 intervention group were analyzed by immunoblotting. **(C)** Immunoblotting analyzed the expression levels of Integrin α5, Integrin β3, SP1 after PBS, Ctrl-R, and JP1 intervention in MEWO cells. **(D, E)** The representative images and quantitative data of Colony formation **(D)** and transwell assays **(E)** of MEWO cells, respectively. n = 3; ***$P < 0.001$. **(F)** The relative SP1 mRNA level was determined by quantitative RT-PCR. **(G, H) (G)** SP1 stability assay. The MEWO cells were treated by JP1 for 48 h and followed by exposed to CHX for indicated time; expression of SP1 was determined by Immunoblotting. **(H)** The time-course intensities of the SP1 protein (n = 3). **$P < 0.01$, ***$P < 0.001$. **(I)** SP1 ubiquitination degradation assay. The MEWO cells were treated JP1 for 48 h followed by MG132 for 6 h; SP1 was determined by Immunoblotting.

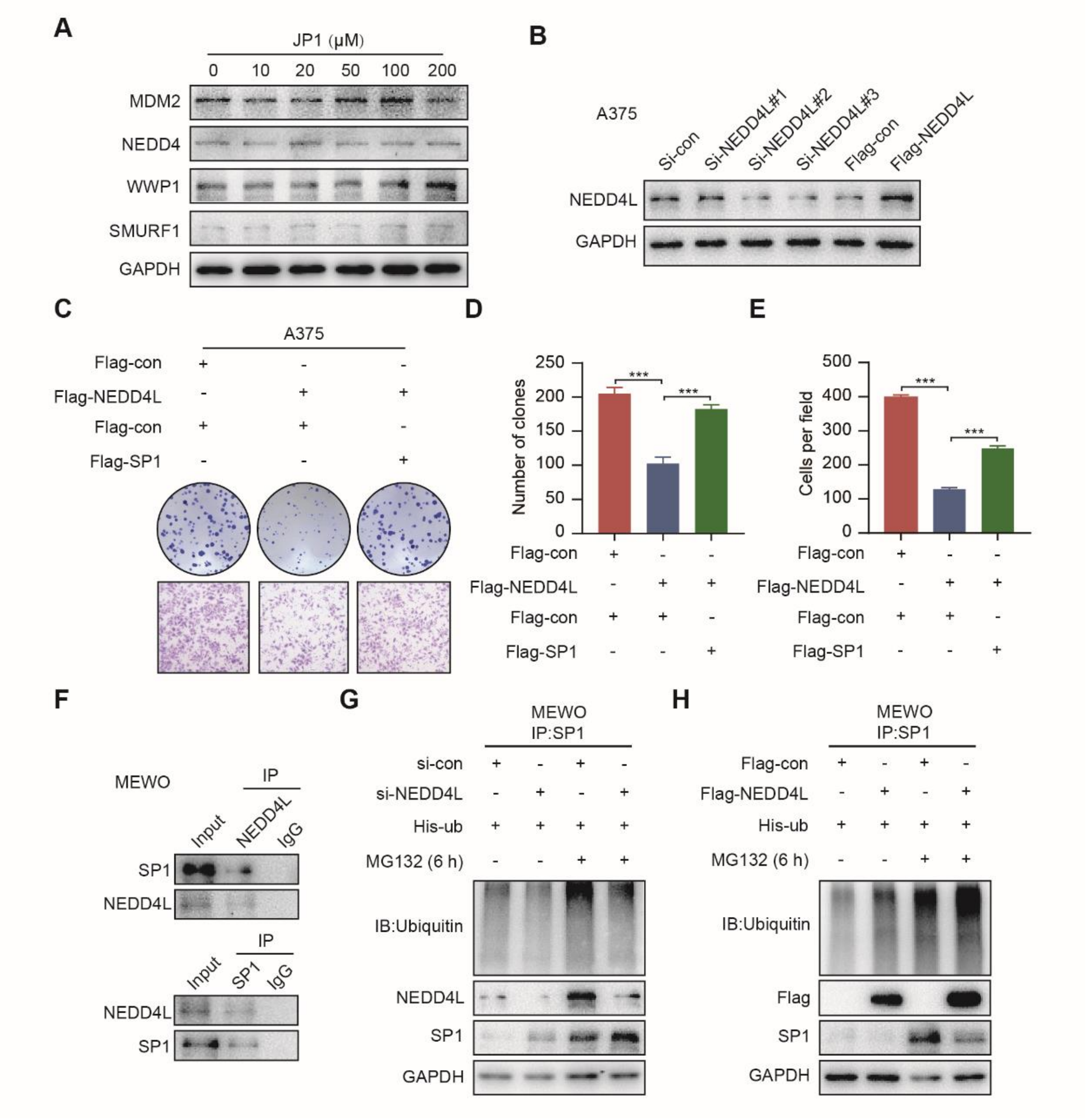

Figure S6. E3 ubiquitin ligase NEDD4L inhibits melanoma cell proliferation and migration through ubiquitination of SP1

(A) The expression of MDM2, NEDD4, WWP1, and SMURF1 after JP1 treatment in A375 cells, Immunoblotting data. **(B)** The expression of NEDD4L after transfected with the NEDD4L-expressing plasmid or vector, and NEDD4L siRNAs (si-NEDD4L#1, si-NEDD4L#2, and si-NEDD4L#3) or si-con in A375 cells, Immunoblotting data. **(C-E)** The colony formation **(C-upper, D)** and transwell assays **(C-lower, E)** for A375 cells transfected NEDD4L-expressing and SP1-expressing plasmid, respectively; n = 3. ****P* < 0.001. **(F)** Analysis of the interactions between NEDD4L and SP1 in MEWO cells; by Immunoblotting. **(G and H)** NEDD4L increases ubiquitination and degradation of SP1 in MEWO cells; by Immunoblotting.

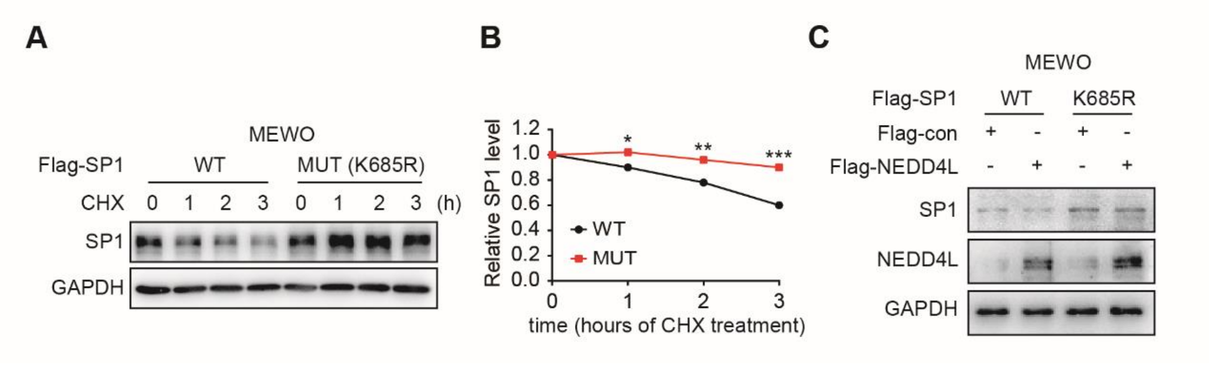

Figure S7. Mutation of K685 site in SP1 prevents its ubiquitination

(A, B) The expressions **(A)** and time course levels **(B)** of Flag-SP1 (WT or K685R) in MEWO cells; by Immunoblotting. n = 3; $*P < 0.05$, $**P < 0.01$, $***P < 0.001$. **(C)** The interactions between Flag-NEDD4L and Flag-SP1 (WT or K685R) in MEWO cells; by Immunoblotting.

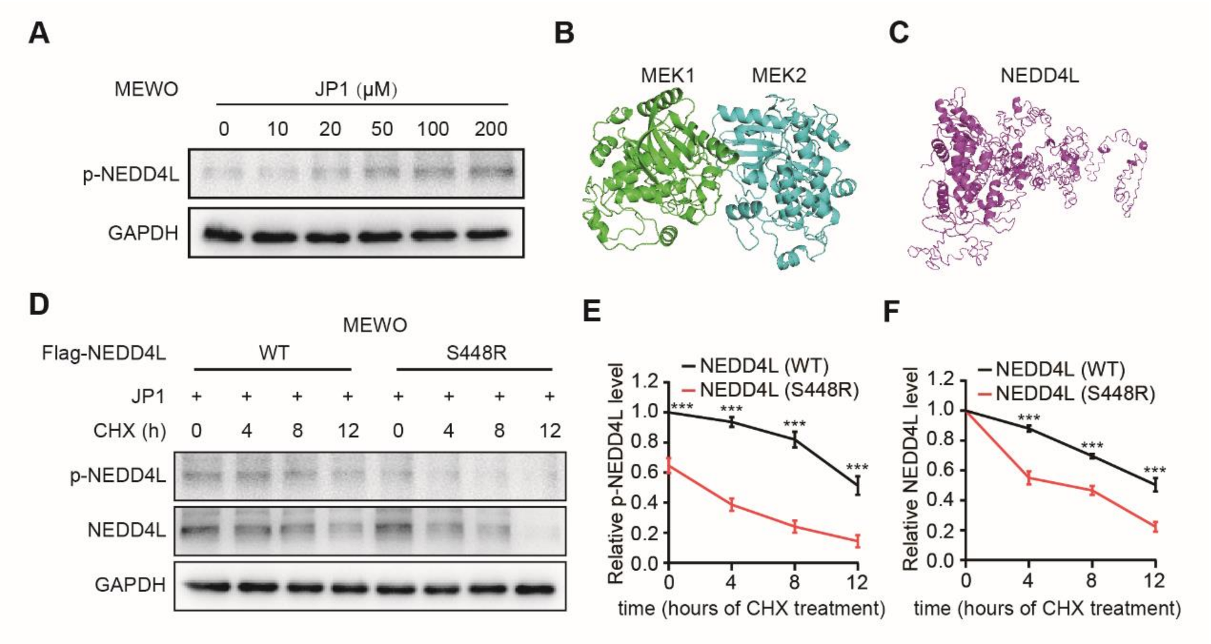

Figure S8. JP1 stabilizes NEDD4L protein expression by activating p-NEDD4L

(A) JP1 dose-dependently induced expressions of p-NEDD4L in MEWO cells; by Immunoblotting. **(B)** The protein structure of MEK1/2. **(C)** The protein structure of NEDD4L. **(D-F)** **(D)** The effects of JP1 on the stability of NEDD4L (WT or S448R) in MEWO cells; by Immunoblotting. The time-course intensities of the p-NEDD4L **(E)** and NEDD4L **(F)** in MEWO cells after transfection of NEDD4L (WT or S448R); n = 3. ****P* < 0.001.

A

Time (h)	JP1-IP-100 mg/kg<br>Conc (ng/mL)		
	201	202	Mean
Pre-dose	NA	NA	NA
0.083	8222.464	17006.67	12614.57
0.25	10421.02	30483.10	20452.06
0.5	15629.99	17975.74	16802.87
1	15648.53	11930.85	13789.69
2	5102.94	3136.05	4119.50
4	44.14	25.51	34.82
8	BLQ	BLQ	NA
24	BLQ	BLQ	NA

BLQ: Below the limit of quantitation
NA: Not applicable

B

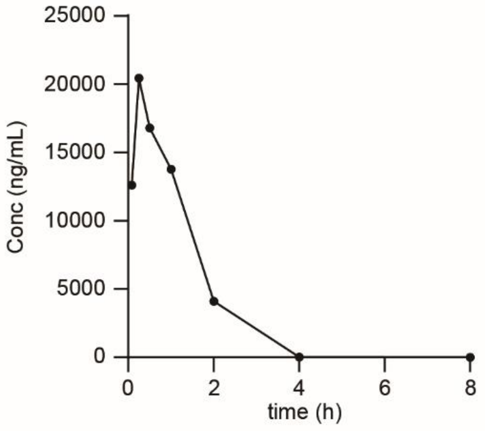

Figure S9. Pharmacokinetic of JP1 in SD rats

(A) The table of drug concentrations at various time points after JP1 treatment. **(B)** The mean drug concentration-time curve of JP1 treatment in SD rats.

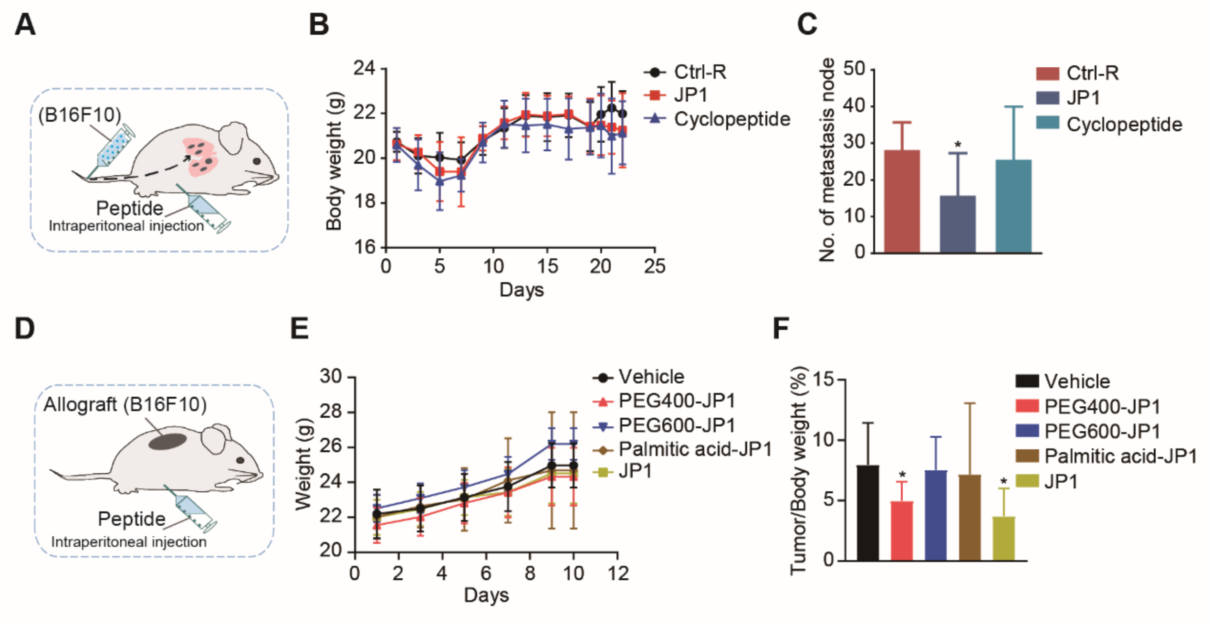

Figure S10. JP1-based modifications of peptides do not show inhibition of the malignant phenotype of melanoma *in vivo*

(A-C) (A) Schematic representation of the B16F10 cell melanoma passive metastasis model for JP1 and cyclopeptide treatment. JP1 and cyclopeptide were administered by intraperitoneal injection. The body weights **(B)** and number of metastasis node per mouse **(C)** were counted after Ctrl-R, JP1 or cyclopeptide treatments. *$P < 0.05$. **(D-E) (D)** Schematic representation of the B16F10 cell melanoma-bearing model for JP1, PEG400-JP1, PEG600-JP1, Palmitic acid-JP1 treatment. JP1 and its modified peptides were administered by intraperitoneal injection. The body weights **(E)** and tumor/body weight ratio **(F)** in Vehicle, PEG400-JP1, PEG600-JP1, Palmitic acid-JP1 or JP1 treated mouse. *$P < 0.05$.

Table S1. The interaction sites of MEK1 and MEK2

MEK1	Interaction	MEK2
ALA14	VDW	PHE319
ASP217	VDW	PHE319
ALA220	VDW	PHE319
ASN221	VDW	PHE319
ALA14	VDW	ILE318
ASN21	VDW	PRO304
GLN214	HBOND	ARG231
LYS185	VDW	PHE227
LYS185	HBOND	SER226
SER212	HBOND	ASN225
GLU114	VDW	ALA224
ARG113	HBOND	ASP221
ARG113	VDW	ASP221
LEU30	VDW	GLU106
ALA26	VDW	HIS104
THR28	VDW	HIS104
LYS183	VDW	GLU106
HIS184	VDW	GLU106
LYS185	VDW	HIS104
LYS185	VDW	GLU106

Table S2. The interaction sites of JP1 and MEK2

JP1	Interaction	MEK2
ASP	IONIC	LYS163
GLY	VDW	LYS160
ARG	VDW	LYS160
GLY	VDW	LYS160
ASP	VDW	GLY307

Table S3. The interaction sites of MEK1 and NEDD4L

MEK1	Interaction	NEDD4L
PRO307	VDW	TYR660
ALA309	VDW	LEU658
GLU312	VDW	LEU658
ALA309	VDW	GLY657
ILE310	HBOND	LYS656
PRO296	VDW	GLU653
ARG305	HBOND	GLU651
PRO307	VDW	TRP649
ARG305	VDW	LYS621
SER304	VDW	ARG537
ARG305	HBOND	ARG537
ARG305	VDW	ARG537
PRO306	VDW	ARG537
ARG305	VDW	HIS535
PRO306	VDW	HIS535
PRO307	VDW	HIS535
SER222	HBOND	LYS489
SER222	VDW	LYS489
ASN221	VDW	PRO488
ASN221	HBOND	SER487
ASN221	VDW	SER487
SER222	HBOND	SER487
PHE223	VDW	SER487
SER222	VDW	ASN486
LYS104	HBOND	TYR485
LYS3	VDW	ARG443
GLY79	VDW	ILE441
VAL81	VDW	ILE441
LEU98	VDW	GLU438
VAL81	VDW	ILE437
PHE83	VDW	ILE437
ASP65	VDW	THR430
ASP65	VDW	ALA429
ILE71	VDW	ARG409
SER72	VDW	ARG409
THR55	VDW	ARG364
GLN56	VDW	ARG364
PRO384	VDW	ASP204
PRO384	VDW	LEU206
ASN382	VDW	THR209
PRO384	VDW	THR209
THR386	VDW	THR209
HIS389	VDW	THR209
GLN383	VDW	TYR211
PRO387	VDW	PRO359
ALA391	VDW	GLY361
ALA391	VDW	ARG362
ALA391	VDW	ALA363

Supplementary database files: Cynomolgus monkey toxicity test

Materials and methods

The study was conducted by a contract service in Suzhou Xishan Zhongke Drugs Research and Development Co., Ltd. (Xishan, Suzhou, China). All the use of animals were approved by the Institute Animal Care and Use Commttee, IACUC. IACUC no. : ID19012912. The veterinarian will supervise the breeding and welfare of the animals. The company follows the Guide for the Care and Use of Laboratory Animals (8th ed., 2011). Cynomolgus monkeys (3-4 years old) were randomly divided into three groups (n = 4 per group, two males and two females for each group); and treated q.d./i.v. with Mannitol (75 mg/kg/d in saline), JP1 (45 mg/kg/d) and JP1 (150 mg/kg/d) for 14 days, respectively. JP1 was dissolved in saline (100 mg/2 ml) and administered intravenously at a rate of 3 ml/min. The JP1 was provided by Hybio Pharmaceutical Co., Ltd. (Shenzhen, China); the production of small packaging preparation (100 mg/vial in freeze-dried powder, purity > 98%; -20 °C for storage and transportation) was completed under strict GMP conditions and quality control procedures for biological preparations. No recovery period was set for this test; the experimental model was ended on 15th day. The relevant clinical indicators including symptoms, body weight, food intake, temperature, ophthalmology, electrocardiogram, blood routine examination, blood biochemistry, blood coagulation, urinalysis, immunotoxicity were determined on 7th and 13th, respectively. No significant changes were observed in the above indicators in all animals, and the pathological observation of organs was not determined.

Conclusion: The 14-day cynomolgus monkey toxicity study showed that all of the relevant clinical indicators in two JP1 groups were no difference compared with those in control group. Therefore, the preliminary data indicate that No Observed Adverse Effect Level (NOAEL) of JP1 was 150 mg/kg.

Table S4. Body weight

Dose (JP1)	Number	Gender	D0	D7	D14
			kg	kg	kg
0 mg/kg	1001	♂	2.5	2.4	2.5
	1002	♂	2.3	2.1	2.2
	2003	♀	2.5	2.3	2.4
	2004	♀	2.3	2.2	2.2
45 mg/kg	1105	♂	2.3	2.1	2.5
	1106	♂	2.4	2.2	2.4
	2107	♀	2.3	2.3	2.4
	2108	♀	2.2	2.1	2.5
150 mg/kg	1209	♂	2.2	2.3	2.2
	1210	♂	2.6	2.4	2.3
	2211	♀	2.3	2.2	2.2
	2212	♀	2.5	2.4	2.6

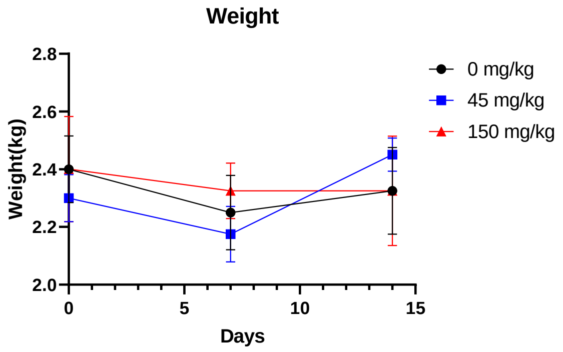

Table S5-1. Blood routine examination

Dose (JP1)	Number	Gender	WBC	NEUT#	LYMPH#	MONO#	EO#	BASO#
**D0**								
			$10^9/L$	$10^9/L$	$10^9/L$	$10^9/L$	$10^9/L$	$10^9/L$
0 mg/kg	1001	♂	11.03	4.58	5.86	0.52	0.07	0.00
	1002	♂	13.52	4.83	7.62	0.68	0.37	0.02
	2003	♀	9.53	5.50	3.32	0.53	0.18	0.00
	2004	♀	33.94	26.56	5.91	1.25	0.21	0.01
45 mg/kg	1105	♂	10.54	2.04	7.22	0.48	0.79	0.01
	1106	♂	12.71	3.14	8.88	0.49	0.18	0.02
	2107	♀	9.47	3.77	5.07	0.50	0.13	0.00
	2108	♀	17.12	8.10	7.17	1.20	0.64	0.01
150 mg/kg	1209	♂	11.82	3.00	7.84	0.74	0.22	0.02
	1210	♂	12.47	1.59	9.58	0.64	0.65	0.01
	2211	♀	9.67	3.52	5.36	0.64	0.14	0.01
	2212	♀	22.17	12.62	7.81	1.54	0.18	0.02
**D7**								
**Dose (JP1)**	**Number**	**Gender**	**WBC**	**NEUT#**	**LYMPH#**	**MONO#**	**EO#**	**BASO#**
			$10^9/L$	$10^9/L$	$10^9/L$	$10^9/L$	$10^9/L$	$10^9/L$
0 mg/kg	1001	♂	10.10	8.16	1.67	0.27	0.00	0.00
	1002	♂	13.82	10.75	2.70	0.34	0.01	0.02
	2003	♀	8.50	6.24	1.64	0.59	0.02	0.01
	2004	♀	31.64	28.87	2.06	0.54	0.16	0.01
45 mg/kg	1105	♂	15.04	12.27	2.38	0.30	0.08	0.01
	1106	♂	10.87	7.64	2.90	0.28	0.04	0.01
	2107	♀	11.55	9.22	2.07	0.25	0.00	0.01
	2108	♀	18.07	11.67	5.56	0.68	0.16	0.00
150 mg/kg	1209	♂	15.42	11.48	3.39	0.53	0.01	0.01
	1210	♂	19.12	14.75	3.63	0.65	0.08	0.01
	2211	♀	12.48	7.71	4.19	0.50	0.07	0.01
	2212	♀	16.18	13.42	2.35	0.39	0.01	0.01
**D13**								
**Dose (JP1)**	**Number**	**Gender**	**WBC**	**NEUT#**	**LYMPH#**	**MONO#**	**EO#**	**BASO#**
			$10^9/L$	$10^9/L$	$10^9/L$	$10^9/L$	$10^9/L$	$10^9/L$
0 mg/kg	1001	♂	6.76	3.24	3.12	0.32	0.07	0.01
	1002	♂	15.13	9.01	5.16	0.69	0.26	0.01
	2003	♀	8.63	4.54	3.47	0.37	0.24	0.01
	2004	♀	16.00	12.66	2.87	0.36	0.10	0.01
45 mg/kg	1105	♂	4.94	2.58	1.95	0.35	0.06	0.00
	1106	♂	9.33	5.01	3.80	0.33	0.18	0.01
	2107	♀	10.10	6.77	2.85	0.44	0.03	0.01
	2108	♀	14.01	7.00	5.98	0.67	0.35	0.01
150 mg/kg	1209	♂	9.44	4.90	4.07	0.39	0.08	0.00
	1210	♂	12.65	6.40	5.06	0.99	0.19	0.01
	2211	♀	8.41	5.17	2.56	0.53	0.14	0.01
	2212	♀	16.30	10.75	4.46	1.04	0.05	0.00

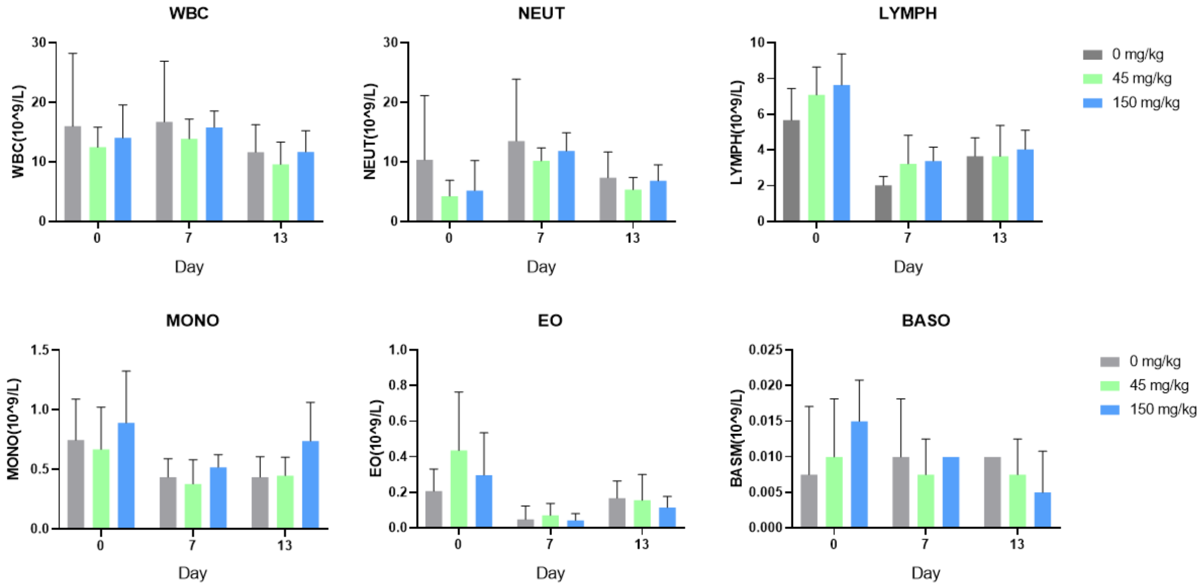

WBC：Leukocyte, NEUT：Neutrophil, LYMPH：Lymphocyte, MONO：Monocyte, EO：Eosinophil, BASO：Basophils

Table S5-2. Blood routine examinations

D0									
**Dose (JP1)**	**Number**	**Gender**	**NEUT %**	**LYMPH %**	**MONO %**	**EO %**	**BASO %**	**RBC**	**HGB**
			**%**	**%**	**%**	**%**	**%**	**10^{12}/L**	**g/L**
0 mg/kg	1001	♂	41.6	53.1	4.7	0.6	0.0	5.34	129
	1002	♂	35.8	56.4	5.0	2.7	0.1	6.64	149
	2003	♀	57.7	34.8	5.6	1.9	0.0	5.18	130
	2004	♀	78.3	17.4	3.7	0.6	0.0	5.12	130
45 mg/kg	1105	♂	19.3	68.5	4.6	7.5	0.1	5.43	127
	1106	♂	24.6	69.9	3.9	1.4	0.2	5.72	133
	2107	♀	39.8	53.5	5.3	1.4	0.0	5.43	132
	2108	♀	47.3	41.9	7.0	3.7	0.1	6.05	143
150 mg/kg	1209	♂	25.3	66.3	6.3	1.9	0.2	5.83	142
	1210	♂	12.8	76.8	5.1	5.2	0.1	6.02	141
	2211	♀	36.5	55.4	6.6	1.4	0.1	5.74	139
	2212	♀	57.0	35.2	6.9	0.8	0.1	5.92	129
**D7**									
**Dose (JP1)**	**Number**	**Gender**	**NEUT %**	**LYMPH %**	**MONO %**	**EO %**	**BASO %**	**RBC**	**HGB**
			**%**	**%**	**%**	**%**	**%**	**10^{12}/L**	**g/L**
0 mg/kg	1001	♂	80.8	16.5	2.7	0.0	0.0	4.65	113
	1002	♂	77.8	19.5	2.5	0.1	0.1	5.52	124
	2003	♀	73.5	19.3	6.9	0.2	0.1	4.49	113
	2004	♀	91.3	6.5	1.7	0.5	0.0	4.39	111
45 mg/kg	1105	♂	81.6	15.8	2.0	0.5	0.1	4.48	104
	1106	♂	70.2	26.7	2.6	0.4	0.1	5.10	118
	2107	♀	79.8	17.9	2.2	0.0	0.1	4.53	111
	2108	♀	64.5	30.8	3.8	0.9	0.0	5.08	119
150 mg/kg	1209	♂	74.4	22.0	3.4	0.1	0.1	4.79	117
	1210	♂	77.1	19.0	3.4	0.4	0.1	4.81	112
	2211	♀	61.7	33.6	4.0	0.6	0.1	4.70	110
	2212	♀	82.9	14.5	2.4	0.1	0.1	4.86	106
**D13**									
**Dose (JP1)**	**Number**	**Gender**	**NEUT %**	**LYMPH %**	**MONO %**	**EO %**	**BASO %**	**RBC**	**HGB**
			**%**	**%**	**%**	**%**	**%**	**10^{12}/L**	**g/L**
0 mg/kg	1001	♂	48.0	46.2	4.7	1.0	0.1	4.82	116
	1002	♂	59.5	34.1	4.6	1.7	0.1	5.64	126
	2003	♀	52.6	40.2	4.3	2.8	0.1	4.47	104
	2004	♀	79.1	17.9	2.3	0.6	0.1	4.52	115
45 mg/kg	1105	♂	52.2	39.5	7.1	1.2	0.0	4.79	121
	1106	♂	53.8	40.7	3.5	1.9	0.1	4.90	113
	2107	♀	67.0	28.2	4.4	0.3	0.1	4.81	115
	2108	♀	49.9	42.7	4.8	2.5	0.1	5.45	127
150 mg/kg	1209	♂	52.0	43.1	4.1	0.8	0.0	4.93	121
	1210	♂	50.6	40.0	7.8	1.5	0.1	4.66	111
	2211	♀	61.5	30.4	6.3	1.7	0.1	4.60	109
	2212	♀	65.9	27.4	6.4	0.3	0.0	5.37	115

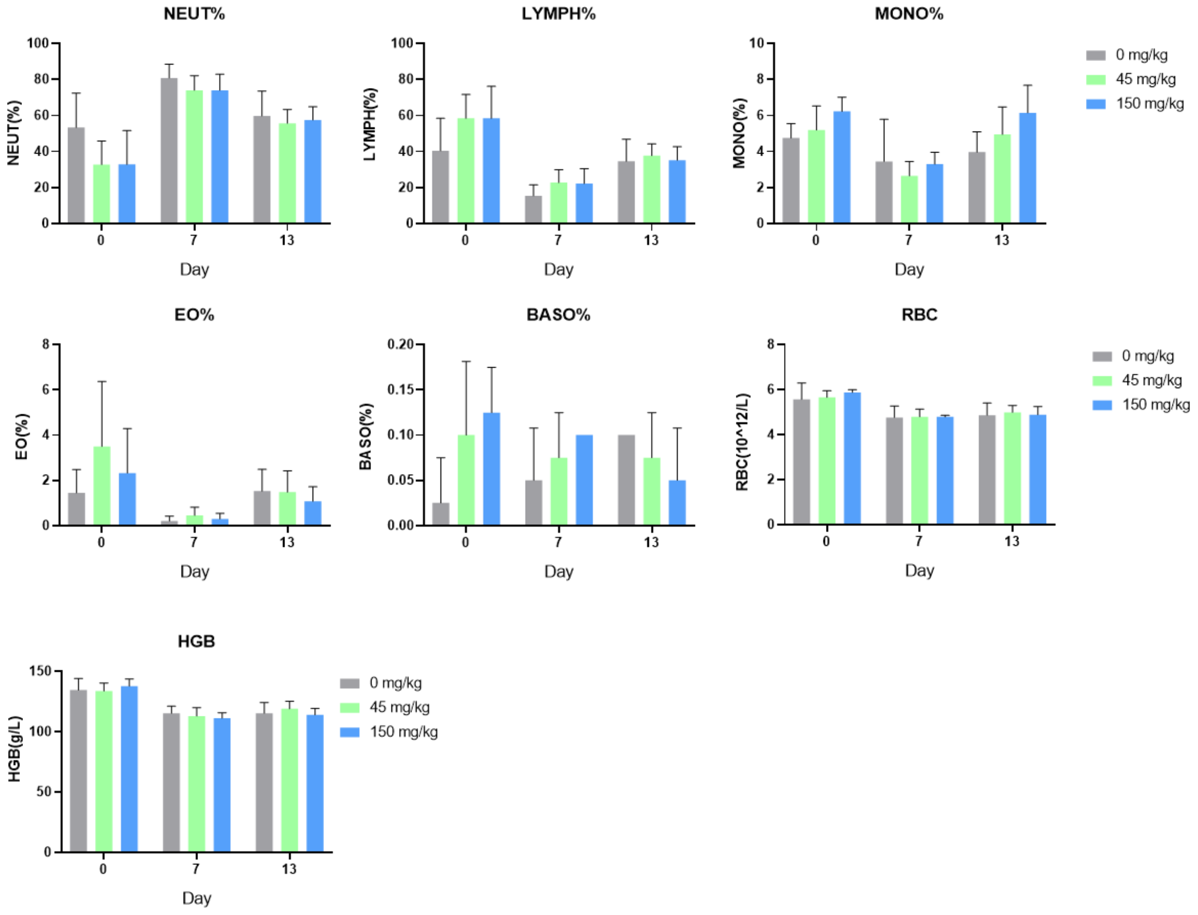

NEUT%：Neutrophil%, LYMPH%：Lymphocyte%, MONO%：Monocyte%, EO%：Eosinophil%, BASO%：Basophils%, RBC：Erythrocyte, HGB：Hemoglobin

Table S5-3. Blood routine examinations (cont.)

Dose (JP1)	Number	Gender	HCT	MCV	MCH	MCHC	PLT	RET%	RET#
**D0**									
			%	fL	pg	g/L	10^9/L	%	10^9/L
0 mg/kg	1001	♂	42.8	80.1	24.2	301	302	1.22	65.1
	1002	♂	46.2	69.6	22.4	323	476	0.50	33.2
	2003	♀	43.3	83.6	25.1	300	352	1.23	63.7
	2004	♀	42.6	83.2	25.4	305	501	0.82	42.0
45 mg/kg	1105	♂	41.4	76.2	23.4	307	332	0.57	31.0
	1106	♂	43.0	75.2	23.3	309	402	0.33	18.9
	2107	♀	43.5	80.1	24.3	303	570	0.81	44.0
	2108	♀	46.6	77.0	23.6	307	507	0.93	56.3
150 mg/kg	1209	♂	44.5	76.3	24.4	319	447	0.91	53.1
	1210	♂	46.7	77.6	23.4	302	380	0.35	21.1
	2211	♀	45.7	79.6	24.2	304	396	0.39	22.4
	2212	♀	42.3	71.5	21.8	305	505	1.24	73.4
**D7**									
**Dose (JP1)**	**Number**	**Gender**	**HCT**	**MCV**	**MCH**	**MCHC**	**PLT**	**RET%**	**RET#**
			%	fL	pg	g/L	10^9/L	%	10^9/L
0 mg/kg	1001	♂	37.3	80.2	24.3	303	294	3.06	142.3
	1002	♂	39.0	70.7	22.5	318	445	1.41	77.8
	2003	♀	38.1	84.9	25.2	297	430	4.35	195.3
	2004	♀	36.5	83.1	25.3	304	477	2.13	93.5
45 mg/kg	1105	♂	34.5	77.0	23.2	301	393	2.67	119.6
	1106	♂	37.9	74.3	23.1	311	476	0.68	34.7
	2107	♀	36.0	79.5	24.5	308	539	2.83	128.2
	2108	♀	39.6	78.0	23.4	301	485	1.78	90.4
150 mg/kg	1209	♂	37.0	77.2	24.4	316	457	0.92	44.1
	1210	♂	38.5	80.0	23.3	291	540	1.47	70.7
	2211	♀	37.2	79.1	23.4	296	391	1.74	81.8
	2212	♀	34.6	71.2	21.8	306	474	1.53	74.4
**D13**									
**Dose (JP1)**	**Number**	**Gender**	**HCT**	**MCV**	**MCH**	**MCHC**	**PLT**	**RET%**	**RET#**
			%	fL	pg	g/L	10^9/L	%	10^9/L
0 mg/kg	1001	♂	39.0	80.9	24.1	297	322	4.90	236.2
	1002	♂	39.8	70.6	22.3	317	477	2.17	122.4
	2003	♀	34.7	77.6	23.3	300	466	3.29	147.1
	2004	♀	37.9	83.8	25.4	303	568	2.43	109.8
45 mg/kg	1105	♂	40.1	83.7	25.3	302	449	3.65	174.8
	1106	♂	36.5	74.5	23.1	310	489	0.95	46.6
	2107	♀	39.2	81.5	23.9	293	561	3.41	164.0
	2108	♀	42.2	77.4	23.3	301	590	1.99	108.5
150 mg/kg	1209	♂	38.3	77.7	24.5	316	451	2.58	127.2
	1210	♂	37.7	80.9	23.8	294	510	1.36	63.4
	2211	♀	36.9	80.2	23.7	295	357	1.63	75.0
	2212	♀	38.8	72.3	21.4	296	548	2.23	119.8

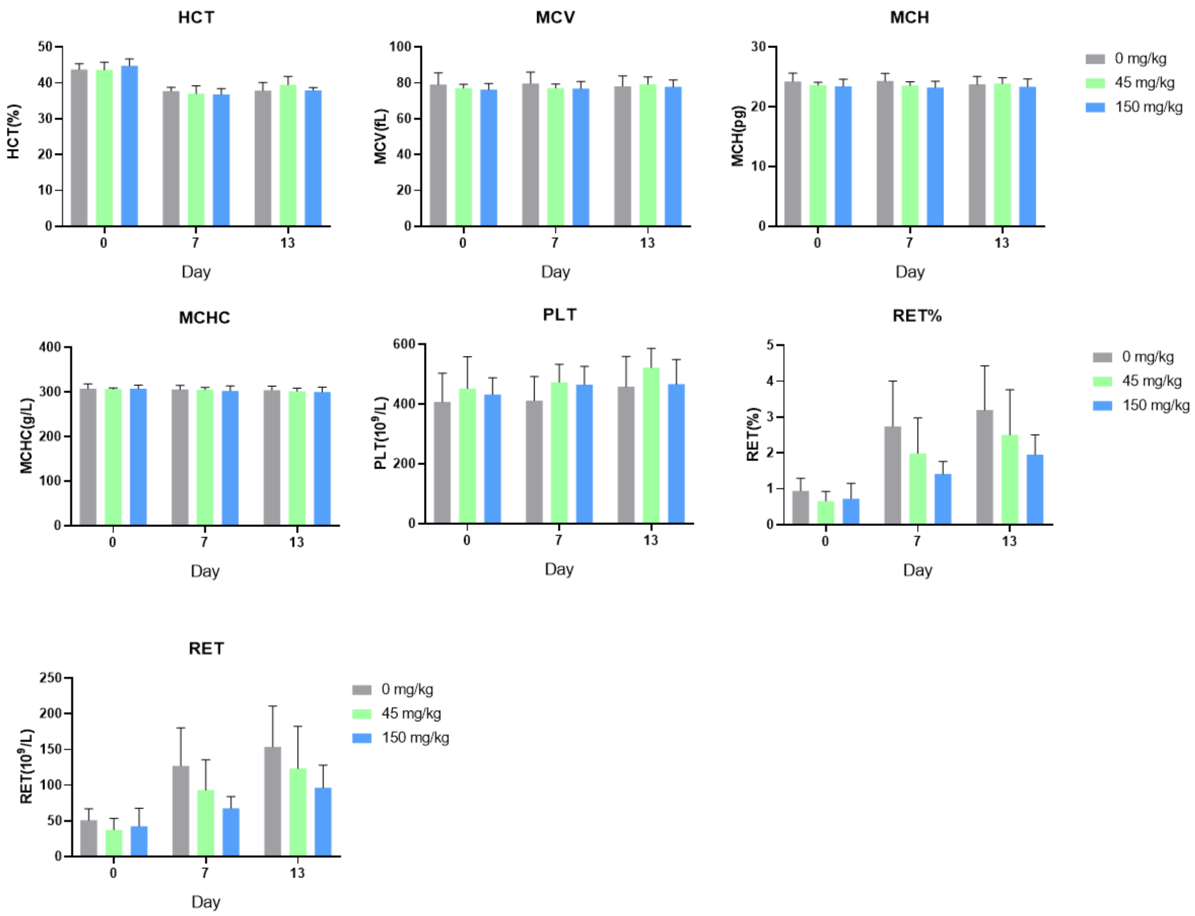

HCT：Hematocrit, MCV：Average red blood cell volume, MCH：Mean corpuscular hemoglobin, MCHC：Mean corpuscular hemoglobin concentration, PLT：Platelet, RET%：Reticulocyte%, RET：Reticulocyte

Table S6-1. Blood biochemistry

Dose (JP1)	Number	Gender	TBIL	TP	ALB	GLOB	A/G	ALT
**D0**								
			μmol/L	g/L	g/L	g/L		U/L
0 mg/kg	1001	♂	1.6	67.6	45.0	22.6	2.0	57
	1002	♂	1.7	79.9	44.7	35.2	1.3	28
	2003	♀	3.3	75.4	45.4	30.0	1.5	32
	2004	♀	1.9	73.0	42.8	30.2	1.4	25
45 mg/kg	1105	♂	1.4	74.9	42.5	32.4	1.3	37
	1106	♂	1.9	72.9	45.0	27.9	1.6	43
	2107	♀	1.4	65.6	40.5	25.1	1.6	27
	2108	♀	1.8	74.2	42.6	31.6	1.3	23
150 mg/kg	1209	♂	2.6	72.3	45.7	26.6	1.7	50
	1210	♂	1.5	71.5	35.8	35.7	1.0	46
	2211	♀	2.1	85.4	46.3	39.1	1.2	37
	2212	♀	2.1	73.5	39.2	34.3	1.1	44
**D7**								
**Dose (JP1)**	**Number**	**Gender**	**TBIL**	**TP**	**ALB**	**GLOB**	**A/G**	**ALT**
			μmol/L	g/L	g/L	g/L		U/L
0 mg/kg	1001	♂	6.8	71.9	48.0	23.9	2.0	74
	1002	♂	3.7	80.4	45.1	35.3	1.3	34
	2003	♀	6.6	75.4	47.0	28.4	1.7	40
	2004	♀	5.0	72.4	44.8	27.6	1.6	37
45 mg/kg	1105	♂	3.3	75.9	45.4	30.5	1.5	63
	1106	♂	4.4	75.8	47.6	28.2	1.7	41
	2107	♀	5.2	67.6	43.0	24.6	1.7	39
	2108	♀	3.6	73.9	43.1	30.8	1.4	27
150 mg/kg	1209	♂	5.0	73.0	47.1	25.9	1.8	62
	1210	♂	3.9	69.9	34.4	35.5	1.0	80
	2211	♀	4.6	79.3	45.6	33.7	1.4	55
	2212	♀	4.9	71.4	40.0	31.4	1.3	36
**D13**								
**Dose (JP1)**	**Number**	**Gender**	**TBIL**	**TP**	**ALB**	**GLOB**	**A/G**	**ALT**
			μmol/L	g/L	g/L	g/L		U/L
0 mg/kg	1001	♂	2.8	68.3	46.6	21.7	2.1	54
	1002	♂	2.1	79.1	44.2	34.9	1.3	26
	2003	♀	6.5	74.2	47.6	26.6	1.8	36
	2004	♀	2.5	71.7	44.7	27.0	1.7	49
45 mg/kg	1105	♂	1.9	72.0	44.0	28.0	1.6	45
	1106	♂	2.3	71.4	45.1	26.3	1.7	38
	2107	♀	2.1	67.7	43.4	24.3	1.8	28
	2108	♀	2.5	74.0	44.2	29.8	1.5	23
150 mg/kg	1209	♂	2.9	73.2	46.8	26.4	1.8	51
	1210	♂	2.6	64.7	32.5	32.2	1.0	46
	2211	♀	2.2	77.5	44.8	32.7	1.4	33
	2212	♀	4.3	72.2	38.5	33.7	1.1	30

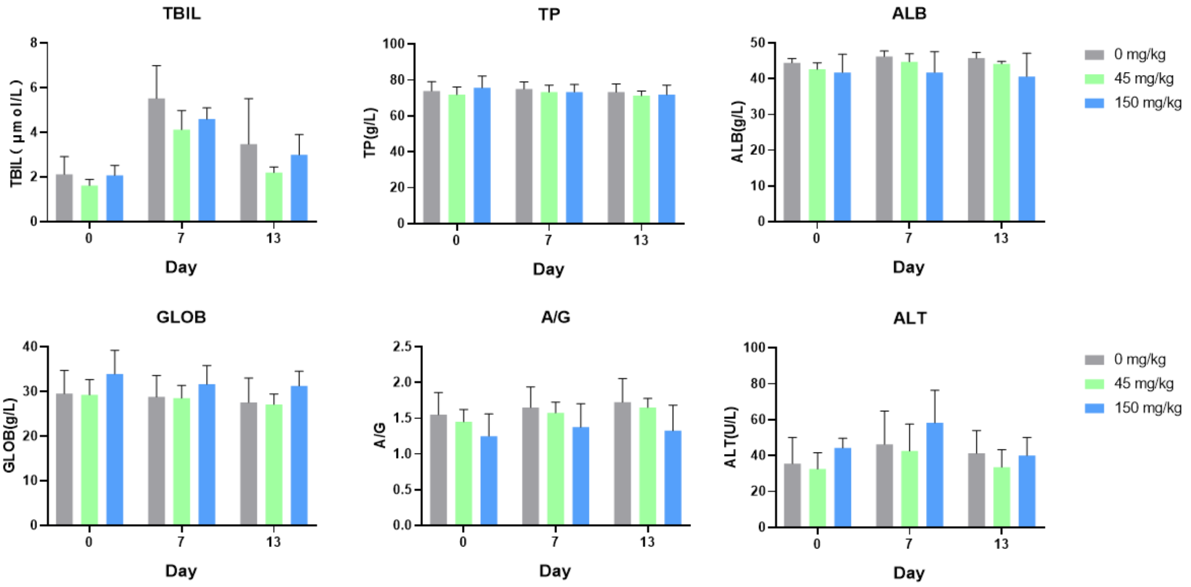

TBIL：Total bilirubin, TP：Total protein, ALB：Albumin, GLAB：Globulin, A/G：Albumin/Globulin, ALT：Alanine aminotransferase

Table S6-2. Blood biochemistry (cont.)

Dose (JP1)	Number	Gender	AST	ALP	GGT	CK	UREA	CREA
**D0**								
			U/L	U/L	U/L	U/L	mmol/L	µmol/L
0 mg/kg	1001	♂	46	589	62	323	7.14	50.2
	1002	♂	23	431	80	176	6.72	54.1
	2003	♀	50	369	58	268	9.41	38.7
	2004	♀	40	429	77	221	8.88	49.4
45 mg/kg	1105	♂	59	539	71	359	7.50	57.4
	1106	♂	48	525	37	246	7.41	53.5
	2107	♀	37	369	39	217	8.53	58.2
	2108	♀	64	488	69	349	8.26	42.0
150 mg/kg	1209	♂	50	418	49	239	8.72	50.4
	1210	♂	65	508	41	460	6.86	45.5
	2211	♀	35	495	77	277	5.93	59.4
	2212	♀	51	778	33	240	9.45	41.9
**D7**								
**Dose (JP1)**	**Number**	**Gender**	**AST**	**ALP**	**GGT**	**CK**	**UREA**	**CREA**
			U/L	U/L	U/L	U/L	mmol/L	µmol/L
0 mg/kg	1001	♂	69	875	64	485	6.12	54.0
	1002	♂	25	546	75	133	8.32	57.6
	2003	♀	46	689	58	153	7.13	37.2
	2004	♀	53	575	79	158	7.74	52.3
45 mg/kg	1105	♂	64	667	71	255	5.60	55.3
	1106	♂	47	614	38	163	6.15	55.1
	2107	♀	53	457	40	291	7.76	60.7
	2108	♀	66	615	68	269	7.59	44.9
150 mg/kg	1209	♂	66	477	50	250	12.09	60.0
	1210	♂	94	765	38	425	6.95	46.4
	2211	♀	45	551	72	237	6.09	49.9
	2212	♀	49	821	31	161	5.97	48.8
**D13**								
**Dose (JP1)**	**Number**	**Gender**	**AST**	**ALP**	**GGT**	**CK**	**UREA**	**CREA**
			U/L	U/L	U/L	U/L	mmol/L	µmol/L
0 mg/kg	1001	♂	46	782	56	289	5.15	50.8
	1002	♂	23	449	70	115	6.77	53.7
	2003	♀	41	514	58	188	6.51	39.9
	2004	♀	53	446	72	128	6.86	46.8
45 mg/kg	1105	♂	53	638	63	231	5.56	55.5
	1106	♂	53	505	33	213	6.33	53.9
	2107	♀	33	396	38	127	6.86	59.0
	2108	♀	60	603	68	295	6.37	45.9
150 mg/kg	1209	♂	47	423	49	204	7.05	52.7
	1210	♂	69	520	34	481	6.54	51.3
	2211	♀	27	471	65	151	5.05	50.3
	2212	♀	53	814	28	374	8.38	49.6

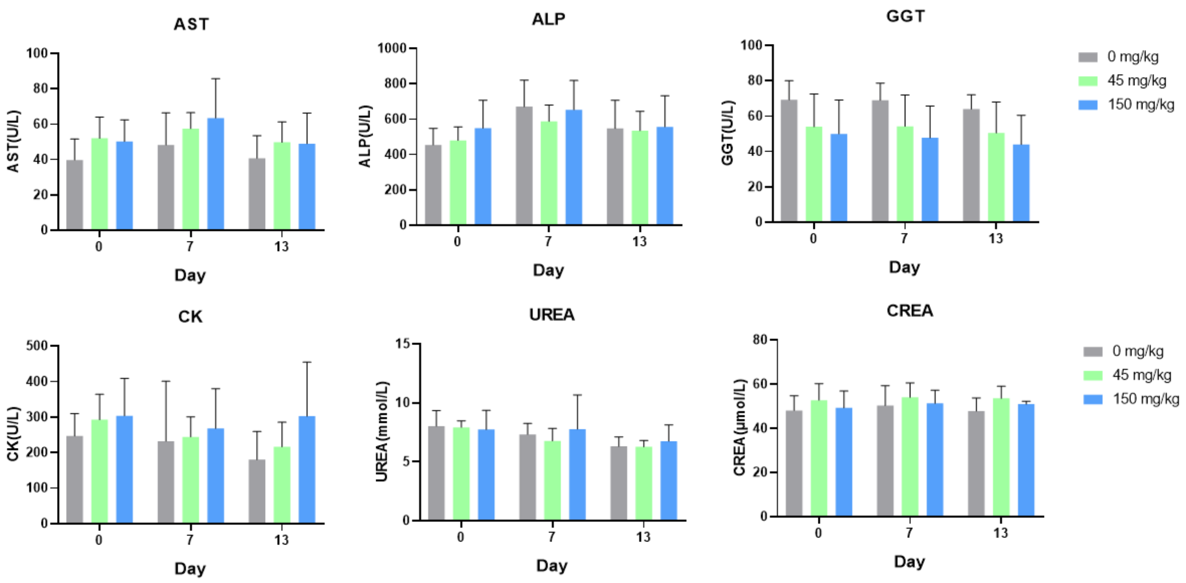

AST：Aspartate aminotransferase, ALP：Alkaline phosphatase, GGT：Glutamic transpeptidase, CK：Creatine kinase, UREA：Serum urea, CREA：Creatinine

Table S6-3. Blood biochemistry (cont.)

D0								
Dose (JP1)	Number	Gender	GLU	TG	CHOL	K^+	Na^+	Cl^-
			mmol/L	mmol/L	mmol/L	mmol/L	mmol/L	mmol/L
0 mg/kg	1001	♂	4.76	0.31	2.59	3.6	144	106
	1002	♂	5.09	0.39	3.67	4.2	149	106
	2003	♀	7.14	0.45	3.64	5.4	146	102
	2004	♀	6.01	0.60	2.94	4.8	144	99
45 mg/kg	1105	♂	6.23	0.53	4.20	4.6	147	102
	1106	♂	5.60	0.27	3.13	5.0	147	101
	2107	♀	7.61	0.48	2.63	4.9	146	107
	2108	♀	3.22	0.74	4.03	4.7	145	100
150 mg/kg	1209	♂	3.77	0.32	3.33	4.0	146	103
	1210	♂	6.58	0.26	2.50	4.6	147	104
	2211	♀	4.79	0.60	2.89	4.1	146	106
	2212	♀	4.34	0.40	3.33	4.2	145	104
D7								
Dose (JP1)	Number	Gender	GLU	TG	CHOL	K^+	Na^+	Cl^-
			mmol/L	mmol/L	mmol/L	mmol/L	mmol/L	mmol/L
0 mg/kg	1001	♂	2.00	0.31	2.65	4.3	141	107
	1002	♂	2.58	0.42	4.25	4.1	143	105
	2003	♀	1.92	0.52	3.72	4.9	143	108
	2004	♀	2.17	0.45	2.79	4.6	141	104
45 mg/kg	1105	♂	2.71	0.31	4.29	4.1	144	109
	1106	♂	3.18	0.23	3.34	4.4	142	104
	2107	♀	2.80	0.42	2.68	4.6	144	110
	2108	♀	3.32	0.41	3.66	4.2	141	105
150 mg/kg	1209	♂	2.80	0.37	3.54	4.3	143	107
	1210	♂	2.04	0.32	2.37	5.8	151	113
	2211	♀	3.28	0.41	2.58	4.4	143	110
	2212	♀	2.45	0.38	3.06	4.3	144	110
D13								
Dose (JP1)	Number	Gender	GLU	TG	CHOL	K^+	Na^+	Cl^-
			mmol/L	mmol/L	mmol/L	mmol/L	mmol/L	mmol/L
0 mg/kg	1001	♂	4.22	0.18	2.83	3.8	143	104
	1002	♂	3.31	0.26	4.01	4.2	145	104
	2003	♀	3.51	0.37	3.95	4.6	143	106
	2004	♀	4.01	0.33	2.63	4.1	143	101
45 mg/kg	1105	♂	4.65	0.28	4.10	4.0	144	104
	1106	♂	4.24	0.17	3.14	4.1	144	103
	2107	♀	4.32	0.41	2.76	5.1	145	107
	2108	♀	3.79	0.54	3.81	4.7	142	102
150 mg/kg	1209	♂	3.65	0.33	3.53	4.3	145	106
	1210	♂	3.46	0.25	1.97	4.3	143	104
	2211	♀	3.84	0.26	2.44	4.2	146	108
	2212	♀	4.61	0.43	3.14	4.4	144	105

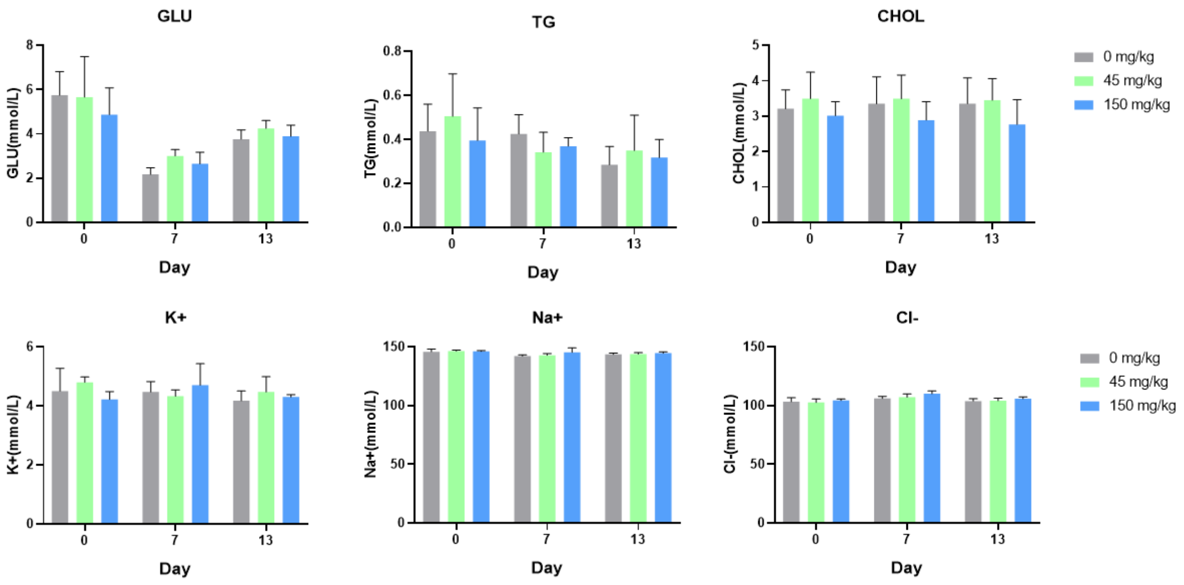

GLU：Fasting blood glucose, TG：Triglyceride, CHOL：Total cholesterol, K+：Serum potassium, Na+：Serum sodium, CI-：Serum chlorine

Table S6-4. Blood biochemistry (cont.)

Dose (JP1)	Number	Gender	CIC	IgG	IgM	C3	C4
**D0**							
			**O.D×100**	**g/L**	**g/L**	**g/L**	**g/L**
0 mg/kg	1001	♂	53.53	4.53	0.53	1.04	0.18
	1002	♂	106.44	9.23	0.88	1.26	0.18
	2003	♀	64.57	7.97	0.81	1.01	0.22
	2004	♀	84.82	7.24	1.39	1.28	0.24
45 mg/kg	1105	♂	94.85	9.10	0.99	1.10	0.17
	1106	♂	85.80	7.39	0.95	0.98	0.17
	2107	♀	59.16	5.59	0.64	1.17	0.22
	2108	♀	97.71	7.46	1.09	1.28	0.31
150 mg/kg	1209	♂	77.08	7.29	0.85	1.14	0.22
	1210	♂	79.11	11.25	1.04	1.06	0.16
	2211	♀	76.36	12.36	0.90	0.43	0.04
	2212	♀	110.68	9.56	0.78	1.04	0.16
**D7**							
**Dose (JP1)**	**Number**	**Gender**	**CIC**	**IgG**	**IgM**	**C3**	**C4**
			**O.D×100**	**g/L**	**g/L**	**g/L**	**g/L**
0 mg/kg	1001	♂	54.28	4.58	0.51	0.94	0.20
	1002	♂	138.68	8.84	0.86	1.33	0.26
	2003	♀	79.90	7.84	0.73	0.95	0.26
	2004	♀	71.88	6.93	1.22	1.01	0.20
45 mg/kg	1105	♂	89.89	8.23	0.78	1.00	0.19
	1106	♂	89.52	7.26	0.89	0.89	0.18
	2107	♀	64.46	5.39	0.57	1.10	0.27
	2108	♀	75.69	7.17	0.93	1.14	0.34
150 mg/kg	1209	♂	81.71	6.50	0.77	1.08	0.21
	1210	♂	83.42	10.42	0.88	1.08	0.17
	2211	♀	66.46	10.16	0.68	0.38	0.04
	2212	♀	94.25	8.62	0.68	0.88	0.16
**D13**							
**Dose (JP1)**	**Number**	**Gender**	**CIC**	**IgG**	**IgM**	**C3**	**C4**
			**O.D×100**	**g/L**	**g/L**	**g/L**	**g/L**
0 mg/kg	1001	♂	59.91	4.37	0.54	1.04	0.21
	1002	♂	125.65	9.16	1.00	1.30	0.22
	2003	♀	77.39	7.83	0.76	0.97	0.23
	2004	♀	60.19	6.79	1.31	1.08	0.19
45 mg/kg	1105	♂	93.29	8.22	0.80	1.00	0.19
	1106	♂	81.11	6.77	0.86	0.94	0.19
	2107	♀	65.73	5.28	0.62	1.20	0.28
	2108	♀	81.00	7.33	1.02	1.06	0.32
150 mg/kg	1209	♂	88.43	6.76	0.86	1.02	0.18
	1210	♂	62.91	10.19	0.91	0.95	0.18
	2211	♀	63.44	10.20	0.72	0.40	0.04
	2212	♀	109.21	9.19	0.93	0.93	0.17

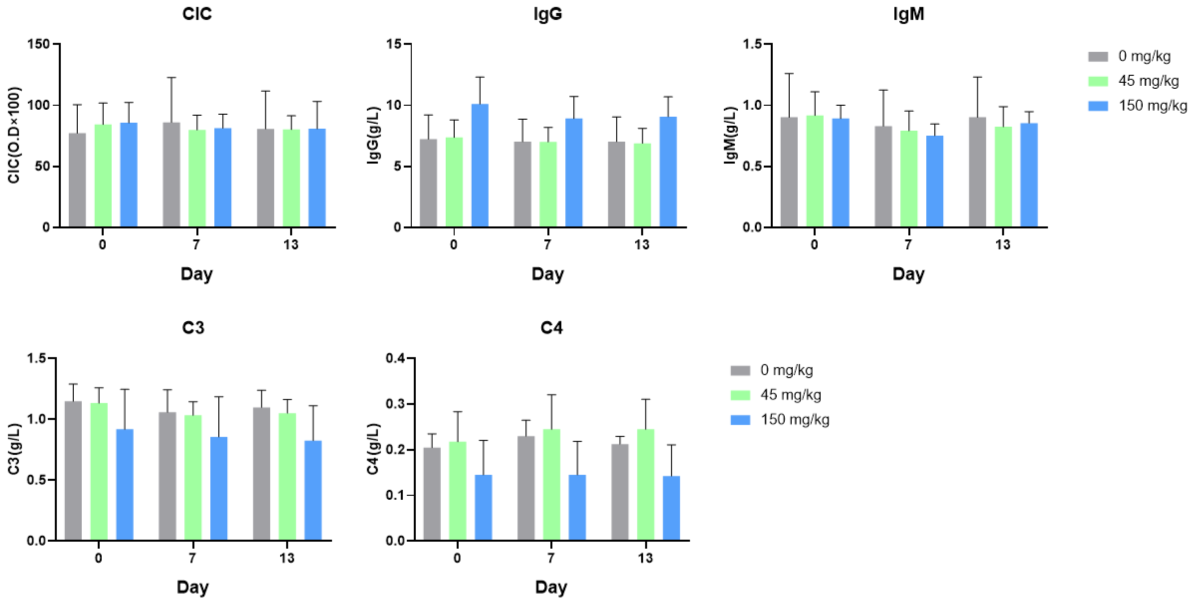

CIC：Circulating immune complex, IgG：Immunoglobulin G, IgM：Immunoglobulin M, C3：Serum complement C3, C4：Serum complement C4

Table S7. Blood coagulation

D0					
Dose (JP1)	Number	Gender	PT	APTT	FIB
			Sec.	Sec.	g/L
0 mg/kg	1001	♂	11.9	29.6	2.25
	1002	♂	11.7	23.6	2.89
	2003	♀	11.5	23.5	2.03
	2004	♀	11.1	24.7	2.92
45 mg/kg	1105	♂	11.4	25.2	2.41
	1106	♂	11.7	24.8	2.61
	2107	♀	11.4	24.7	2.09
	2108	♀	10.9	23.3	2.53
150 mg/kg	1209	♂	11.6	25.1	2.69
	1210	♂	12.7	27.6	2.65
	2211	♀	11.8	33.8	2.62
	2212	♀	12.1	29.7	2.41
D7					
Dose (JP1)	Number	Gender	PT	APTT	FIB
			Sec.	Sec.	g/L
0 mg/kg	1001	♂	12.1	28.8	2.46
	1002	♂	11.8	22.5	4.07
	2003	♀	12.0	23.1	2.33
	2004	♀	10.8	24.8	2.56
45 mg/kg	1105	♂	11.8	28.0	2.84
	1106	♂	12.2	25.5	2.33
	2107	♀	11.9	25.1	2.34
	2108	♀	11.0	23.2	2.59
150 mg/kg	1209	♂	11.8	23.7	2.71
	1210	♂	12.5	25.2	3.21
	2211	♀	11.7	33.8	2.66
	2212	♀	12.0	27.7	2.89
D13					
Dose (JP1)	Number	Gender	PT	APTT	FIB
			Sec.	Sec.	g/L
0 mg/kg	1001	♂	11.5	26.2	2.60
	1002	♂	11.6	22.9	3.38
	2003	♀	11.7	23.3	2.35
	2004	♀	11.1	24.6	2.97
45 mg/kg	1105	♂	11.7	25.3	3.02
	1106	♂	12.2	24.6	2.81
	2107	♀	11.7	24.2	2.68
	2108	♀	10.9	22.6	2.51
150 mg/kg	1209	♂	11.9	23.5	2.90
	1210	♂	12.4	24.9	2.99
	2211	♀	11.8	29.9	2.97
	2212	♀	11.7	27.8	3.27

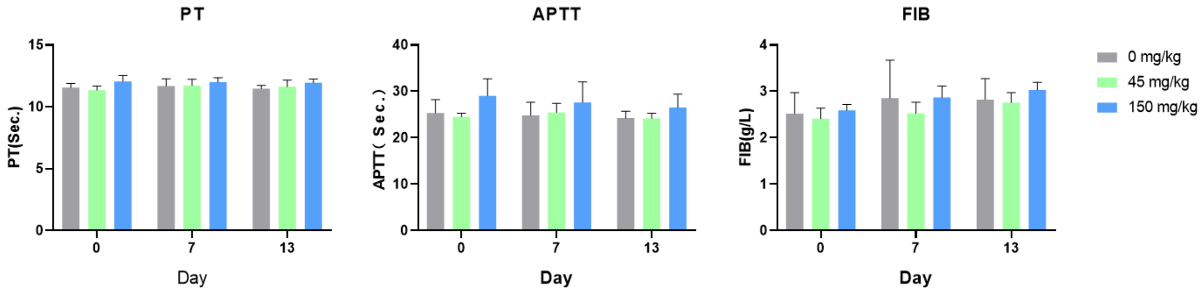

PT：Plasma prothrombin time, APTT：Activated partial thromboplastin time, FIB：Fibrinogen

Table S8-1. Urinalysis

D0							
**Dose (JP1)**	**Number**	**Gender**	**COL**	**TURB**	**pH**	**NIT**	**GLU**
0 mg/kg	1001	♂	LIGHT RED	2	8.5	-	NORMAL
	1002	♂	LIGHT YELLOW	1	7.5	-	NORMAL
	2003	♀	YELLOW	1	8.5	-	3
	2004	♀	YELLOW	1	8.5	-	NORMAL
45 mg/kg	1105	♂	RED	1	6.5	-	NORMAL
	1106	♂	LIGHT YELLOW	1	8	-	NORMAL
	2107	♀	LIGHT YELLOW	1	8	-	NORMAL
	2108	♀	LIGHT YELLOW	1	8.5	-	NORMAL
150 mg/kg	1209	♂	COLORLESS	-	8.5	-	NORMAL
	1210	♂	LIGHT RED	1	8.5	-	NORMAL
	2211	♀	LIGHT RED	1	8	-	NORMAL
	2212	♀	LIGHT RED	2	8.5	-	NORMAL
**D7**							
**Dose (JP1)**	**Number**	**Gender**	**COL**	**TURB**	**pH**	**NIT**	**GLU**
0 mg/kg	1001	♂	COLORLESS	+1	8.5	+1	NORMAL
	1002	♂	COLORLESS	+1	8.0	-	NORMAL
	2003	♀	LIGHT YELLOW	+1	8.5	+1	NORMAL
	2004	♀	COLORLESS	+1	8.5	-	NORMAL
45 mg/kg	1105	♂	COLORLESS	+1	8.0	+1	NORMAL
	1106	♂	YELLOW	+1	8.5	+1	NORMAL
	2107	♀	COLORLESS	+1	8.0	-	NORMAL
	2108	♀	COLORLESS	-	8.5	-	NORMAL
150 mg/kg	1209	♂	LIGHT YELLOW	+1	7.5	-	NORMAL
	1210	♂	YELLOW	+1	8.5	-	NORMAL
	2211	♀	YELLOW	+2	8.5	-	NORMAL
	2212	♀	COLORLESS	+1	8.5	-	NORMAL
**D13**							
**Dose (JP1)**	**Number**	**Gender**	**COL**	**TURB**	**pH**	**NIT**	**GLU**
0 mg/kg	1001	♂	COLORLESS	-	8.5	-	NORMAL
	1002	♂	COLORLESS	-	8.5	-	NORMAL
	2003	♀	COLORLESS	+1	8.0	-	NORMAL
	2004	♀	COLORLESS	-	6.5	-	NORMAL
45 mg/kg	1105	♂	COLORLESS	+1	7.5	-	NORMAL
	1106	♂	COLORLESS	-	8.0	-	NORMAL
	2107	♀	COLORLESS	+1	8.5	-	NORMAL
	2108	♀	COLORLESS	+1	8.0	-	NORMAL
150 mg/kg	1209	♂	COLORLESS	-	8.0	-	NORMAL
	1210	♂	LIGHT YELLOW	+1	8.5	-	NORMAL
	2211	♀	COLORLESS	+1	8.0	-	NORMAL
	2212	♀	COLORLESS	+1	7.5	-	NORMAL

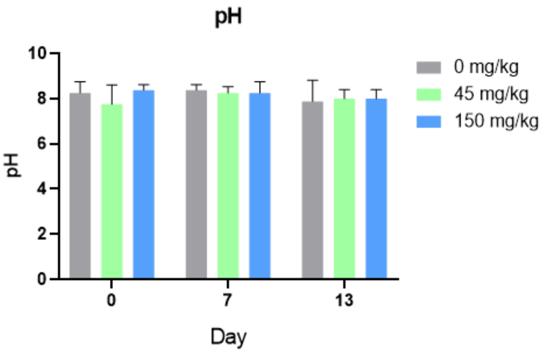

COL：Urine color, TURB：Urine transparency, pH：Urine pH, NIT：Urine nitrite, GLU：Urine glucose

Table S8-2. Urinalysis

Dose (JP1)	Number	Gender	SG	BLD	PRO	URO	KET	LEU (Leu/uL)
**D0**								
0 mg/kg	1001	♂	1.025	2	1	NORMAL	-	NEG.
	1002	♂	1.011	+-	+-	NORMAL	-	NEG.
	2003	♀	1.027	+-	1	NORMAL	-	25
	2004	♀	1.016	+-	1	NORMAL	-	NEG.
45 mg/kg	1105	♂	1.004	+-	-	NORMAL	-	NEG.
	1106	♂	1.010	-	+-	NORMAL	-	NEG.
	2107	♀	1.008	2	-	NORMAL	-	NEG.
	2108	♀	1.016	-	+-	NORMAL	-	25
150 mg/kg	1209	♂	1.005	2	-	NORMAL	-	NEG.
	1210	♂	1.007	-	-	NORMAL	-	NEG.
	2211	♀	1.009	-	+-	NORMAL	-	NEG.
	2212	♀	1.010	1	+-	NORMAL	-	NEG.
**D7**								
**Dose (JP1)**	**Number**	**Gender**	**SG**	**BLD**	**PRO**	**URO**	**KET**	**LEU (Leu/uL)**
0 mg/kg	1001	♂	1.006	-	-	NORMAL	+-	NEG.
	1002	♂	1.002	+3	+-	NORMAL	-	NEG.
	2003	♀	1.009	+-	+-	NORMAL	-	NEG.
	2004	♀	1.007	+3	+-	NORMAL	-	NEG.
45 mg/kg	1105	♂	1.004	-	-	NORMAL	-	NEG.
	1106	♂	1.012	-	+3	NORMAL	-	NEG.
	2107	♀	1.005	+2	-	NORMAL	-	25
	2108	♀	1.008	-	-	NORMAL	-	NEG.
150 mg/kg	1209	♂	1.002	+-	-	NORMAL	-	NEG.
	1210	♂	1.009	-	+1	NORMAL	-	NEG.
	2211	♀	1.020	+1	+1	NORMAL	-	NEG.
	2212	♀	1.006	+1	+-	NORMAL	+-	75
**D13**								
**Dose (JP1)**	**Number**	**Gender**	**SG**	**BLD**	**PRO**	**URO**	**KET**	**LEU (Leu/uL)**
0 mg/kg	1001	♂	1.009	+-	-	NORMAL	-	NEG.
	1002	♂	1.006	-	-	NORMAL	-	NEG.
	2003	♀	1.008	+1	-	NORMAL	-	NEG.
	2004	♀	1.004	-	-	NORMAL	-	NEG.
45 mg/kg	1105	♂	1.006	+1	-	NORMAL	-	25
	1106	♂	1.007	+1	-	NORMAL	-	NEG.
	2107	♀	1.009	-	-	NORMAL	-	NEG.
	2108	♀	1.006	+1	-	NORMAL	-	NEG.
150 mg/kg	1209	♂	1.004	-	-	NORMAL	-	NEG.
	1210	♂	1.013	+1	+-	NORMAL	-	NEG.
	2211	♀	1.005	+3	-	NORMAL	-	NEG.
	2212	♀	1.009	+1	-	NORMAL	-	NEG.

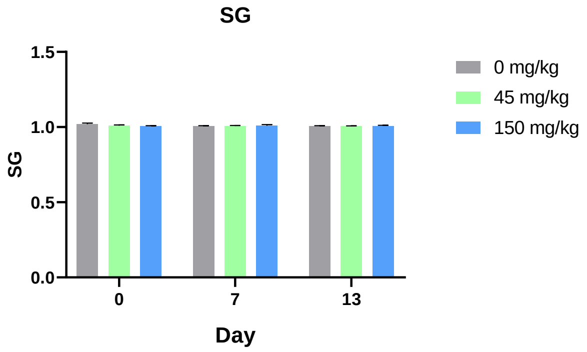

SG：Urine specific gravity, BLD：Hidden blood, PRO：Urinary protein, URO：Urobilinogen, KET：Ketone body, LEU： Urine leukocyte

Table S9. Immunotoxicity

D0					
**Dose (JP1)**	**Number**	**Gender**	**$CD3^+CD4^+$**	**$CD3^+CD8^+$**	**CD4/CD8**
			**%**	**%**	**%**
0 mg/kg	1001	♂	25.400	40.880	1.609
	1002	♂	22.240	42.200	1.897
	2003	♀	21.760	34.740	1.597
	2004	♀	20.520	40.060	1.952
45 mg/kg	1105	♂	14.980	29.960	2.000
	1106	♂	25.720	32.940	1.281
	2107	♀	19.280	42.900	2.225
	2108	♀	13.740	29.680	2.160
150 mg/kg	1209	♂	32.760	33.960	1.037
	1210	♂	35.960	26.840	0.746
	2211	♀	51.220	21.300	0.416
	2212	♀	21.860	30.080	1.376
**D7**					
**Dose (JP1)**	**Number**	**Gender**	**$CD3^+CD4^+$**	**$CD3^+CD8^+$**	**CD4/CD8**
			**%**	**%**	**%**
0 mg/kg	1001	♂	18.720	30.600	1.635
	1002	♂	20.420	46.120	2.259
	2003	♀	23.160	34.680	1.497
	2004	♀	17.740	36.240	2.043
45 mg/kg	1105	♂	14.420	25.500	1.768
	1106	♂	20.160	32.040	1.589
	2107	♀	13.220	35.620	2.694
	2108	♀	14.760	27.140	1.839
150 mg/kg	1209	♂	27.600	35.120	1.272
	1210	♂	28.160	32.940	1.170
	2211	♀	44.860	27.060	0.603
	2212	♀	19.080	33.660	1.764
**D13**					
**Dose (JP1)**	**Number**	**Gender**	**$CD3^+CD4^+$**	**$CD3^+CD8^+$**	**CD4/CD8**
			**%**	**%**	**%**
0 mg/kg	1001	♂	18.703	47.473	2.538
	1002	♂	19.049	48.331	2.537
	2003	♀	21.320	45.980	2.157
	2004	♀	15.040	44.920	2.987
45 mg/kg	1105	♂	13.100	41.820	3.192
	1106	♂	19.100	43.140	2.259
	2107	♀	14.180	47.160	3.326
	2108	♀	13.220	31.220	2.362
150 mg/kg	1209	♂	24.420	40.460	1.657
	1210	♂	20.280	36.280	1.789
	2211	♀	28.260	38.920	1.377
	2212	♀	22.580	46.260	2.049

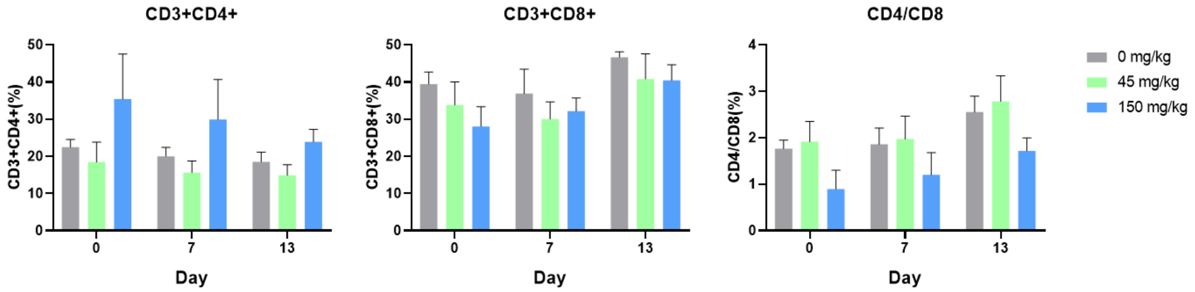
CD3+CD4+
CD3+CD4+(%)
Day
CD3+CD8+
CD3+CD8+(%)
Day
CD4/CD8
CD4/CD8(%)
Day
0 mg/kg
45 mg/kg
150 mg/kg
